# 2023 UPDATE: Luso-Brazilian evidence-based guideline for the management of antidiabetic therapy in type 2 diabetes

**DOI:** 10.1186/s13098-023-01121-x

**Published:** 2023-07-19

**Authors:** Marcello Casaccia Bertoluci, Wellington S. Silva Júnior, Fernando Valente, Levimar Rocha Araujo, Ruy Lyra, João Jácome de Castro, João Filipe Raposo, Paulo Augusto Carvalho Miranda, Cesar Luiz Boguszewski, Alexandre Hohl, Rui Duarte, João Eduardo Nunes Salles, José Silva-Nunes, Jorge Dores, Miguel Melo, João Roberto de Sá, João Sérgio Neves, Rodrigo Oliveira Moreira, Marcus Vinícius Bolívar Malachias, Rodrigo Nunes Lamounier, Domingos Augusto Malerbi, Luis Eduardo Calliari, Luis Miguel Cardoso, Maria Raquel Carvalho, Hélder José Ferreira, Rita Nortadas, Fábio Rogério Trujilho, Cristiane Bauermann Leitão, José Augusto Rodrigues Simões, Mónica Isabel Natal dos Reis, Pedro Melo, Mafalda Marcelino, Davide Carvalho

**Affiliations:** 1 Faculdade de Medicina da Universidade Federal do Rio Grande do Sul, Porto Alegre, Brazil; 2 Serviço de Endocrinologia do Hospital de Clínicas de Porto Alegre, Departamento de Medicina Interna da Universidade Federal do Rio Grande do Sul (UFRGS), Rua Ramiro Barcelos, 2350, 4º Andar, Porto Alegre, RS 90035-007 Brazil; 3 Disciplina de Endocrinologia, Departamento de Medicina I, Universidade Federal Maranhão, São Luís, Brazil; 4 Faculdade de Medicina do ABC, Santo André, Brazil; 5 Faculdade de Ciências Médicas de Minas Gerais, Belo Horizonte, Brazil; 6 Universidade Federal de Pernambuco, Recife, Brazil; 7 Serviço de Endocrinologia do Hospital Universitário das Forças Armadas, Lisbon, Portugal; 8 NOVA Medical School, Universidade Nova de Lisboa, Lisbon, Portugal; 9 Clínica de Endocrinologia e Metabologia da Santa Casa Belo Horizonte, Belo Horizonte, Brazil; 10 Divisão de Endocrinologia (SEMPR), Departamento de Clínica Médica, Universidade Federal do Paraná, Curitiba, Brazil; 11 Departamento de Clínica Médica da Universidade Federal de Santa Catarina, Florianópolis, Brazil; 12 Associação Protectora dos Diabéticos de Portugal, Lisbon, Portugal; 13 Faculdade de Ciências Médicas da Santa Casa de São Paulo, São Paulo, Brazil; 14 Centro Hospitalar e Universitário de Santo António, Lisbon, Portugal; 15 Instituto de Ciências Biomédicas Abel Salazar da Universidade do Porto, Porto, Portugal; 16 Serviço de Endocrinologia, Diabetes e Metabolismo, Centro Hospitalar e Universitário de Coimbra, Faculdade de Medicina da Universidade de Coimbra, Coimbra, Portugal; 17 Escola Paulista de Medicina, Universidade Federal de São Paulo, São Paulo, Brazil; 18 Cardiovascular R&D Centre (UnIC@RISE), Faculdade de Medicina da Universidade do Porto, Porto, Portugal; 19 Serviço de Endocrinologia, Diabetes e Metabolismo, Centro Hospitalar Universitário de São João, Porto, Portugal; 20 Instituto Estadual de Diabetes e Endocrinologia Luiz Capriglione (IEDE), Rio de Janeiro, Brazil; 21 Faculdade de Medicina, Centro Universitário Presidente Antônio Carlos (UNIPAC/JF), Juiz de Fora, Brazil; 22 Faculdade de Medicina, Centro Universitário de Valença (UNIFAA), Valença, Brazil; 23 Departamento de Clínica Médica da Faculdade de Medicina da Universidade Federal de Minas Gerais, Belo Horizonte, Brazil; 24 Hospital Israelita Albert Einstein, São Paulo, Brazil; 25 Hospital CUF, Tejo, Portugal; 26 Clínica Grupo Sanfil Medicina, Coimbra, Portugal; 27 Faculdade de Medicina da UniFTC, Salvador, Brazil; 28 Faculdade de Ciências da Saúde da Universidade da Beira Interior, Covilhã, Portugal; 29 Unidade Integrada de Diabetes Mellitus do Hospital de Vila Franca de Xira, Vila Franca de Xira, Portugal; 30 Serviço de Endocrinologia, Hospital Pedro Hispano, Matosinhos, Portugal; 31 i3S, Instituto de Investigação e Inovação em Saúde, Universidade do Porto, Porto, Portugal; 32 Centro de Diabetes e Endocrinologia da Bahia (CEDEBA), Salvador, Brazil; 33 Serviço de Endocrinologia, Diabetes e Metabolismo, Centro Hospitalar e Universitário de Coimbra, Coimbra, Portugal; 34 Unidade de Endocrinologia, Instituto CUF, Porto, Portugal; 35 Faculdade de Medicina da Universidade do Porto, Porto, Portugal; 36 Sociedade Brasileira de Diabetes (SBD), São Paulo, Brazil; 37 Sociedade Brasileira de Endocrinologia e Metabologia (SBEM), Rio de Janeiro, Brazil; 38 Sociedade Portuguesa de Diabetologia (SPD), Lisbon, Portugal; 39 Sociedade Portuguesa de Endocrinologia, Diabetes e Metabolismo (SPEDM), Lisbon, Portugal

**Keywords:** ASCVD, Atherosclerotic disease, Cardiovascular risk, Chronic kidney disease, DKD, Diabetes treatment, Guidelines, Heart failure, Ischemic heart disease, Type 2 diabetes, SGLT2 inhibitors, GLP-1 RA

## Abstract

**Background:**

The management of antidiabetic therapy in people with type 2 diabetes (T2D) has evolved beyond glycemic control. In this context, Brazil and Portugal defined a joint panel of four leading diabetes societies to update the guideline published in 2020.

**Methods:**

The panelists searched MEDLINE (via PubMed) for the best evidence from clinical studies on treating T2D and its cardiorenal complications. The panel searched for evidence on antidiabetic therapy in people with T2D without cardiorenal disease and in patients with T2D and atherosclerotic cardiovascular disease (ASCVD), heart failure (HF), or diabetic kidney disease (DKD). The degree of recommendation and the level of evidence were determined using predefined criteria.

**Results and conclusions:**

All people with T2D need to have their cardiovascular (CV) risk status stratified and HbA1c, BMI, and eGFR assessed before defining therapy. An HbA1c target of less than 7% is adequate for most adults, and a more flexible target (up to 8%) should be considered in frail older people. Non-pharmacological approaches are recommended during all phases of treatment. In treatment naïve T2D individuals without cardiorenal complications, metformin is the agent of choice when HbA1c is 7.5% or below. When HbA1c is above 7.5% to 9%, starting with dual therapy is recommended, and triple therapy may be considered. When HbA1c is above 9%, starting with dual therapyt is recommended, and triple therapy should be considered. Antidiabetic drugs with proven CV benefit (AD1) are recommended to reduce CV events if the patient is at high or very high CV risk, and antidiabetic agents with proven efficacy in weight reduction should be considered when obesity is present. If HbA1c remains above target, intensification is recommended with triple, quadruple therapy, or even insulin-based therapy. In people with T2D and established ASCVD, AD1 agents (SGLT2 inhibitors or GLP-1 RA with proven CV benefit) are initially recommended to reduce CV outcomes, and metformin or a second AD1 may be necessary to improve glycemic control if HbA1c is above the target. In T2D with HF, SGLT2 inhibitors are recommended to reduce HF hospitalizations and mortality and to improve HbA1c. In patients with DKD, SGLT2 inhibitors in combination with metformin are recommended when eGFR is above 30 mL/min/1.73 m^2^. SGLT2 inhibitors can be continued until end-stage kidney disease.

## Introduction

Treatment of type 2 diabetes mellitus (T2D) has evolved rapidly in recent years. New agents and strategies have amplified the scopus for managing T2D, and much new evidence has emerged. Therefore, the four leading Diabetes Societies from Brazil and Portugal (*Sociedade Brasileira de Diabetes [SBD], Sociedade Brasileira de Endocrinologia e Metabologia [SBEM], Sociedade Portuguesa Diabetologia [SPD], and Sociedade Portuguesa de Endocrinologia, Diabetes e Metabolismo* [SPEDM]) joined to update the initial version of Portuguese-Brazilian guideline on the management of hyperglycemia in T2D, published in 2020 [1]. The panel gathered the best evidence in the field, and a grade of recommendation was established through polls.

### What is new in the 2023 UPDATE?

The 2023 UPDATE brings a paradigm shift from the previous guideline focused on treating hyperglycemia. The new evidence-based recommendations guide the management of antidiabetic therapy and involve aspects beyond glycemic control, such as achieving and maintaining a healthy weight and cardiorenal protection.

Non-pharmacological approaches were revised, and they now include recommendations related to sleep duration, sitting time, and the use of continuous glucose monitoring (CGM). There have been notable updates in the criteria for selecting the most appropriate therapy. For this purpose, the 2023 UPDATE recommends stratifying cardiovascular (CV) risk and defining the weight status, renal function, and glycated hemoglobin (HbA1c) level of all individuals with T2D. The panel included a new table with revised CV risk factors and new CV risk markers of subclinical disease or end-organ lesion, such as N-terminal pro-B-type natriuretic peptide (NT-proBNP) and advanced microvascular complications (proliferative diabetic retinopathy, severe cardiac autonomic neuropathy, and advanced stages of renal disease).

Although pharmacological treatment still includes AD1 (antidiabetic agents with proven CV benefits) and AD (anti-hyperglycemic agents with proven CV safety), the 2023 UPDATE highlights agents with efficacy in weight management, i.e., glucagon-like peptide-1 receptor agonists (GLP-1 RA) and the new class of dual glucose-dependent insulinotropic polypeptide (GIP)/glucagon-like peptide-1 (GLP-1) receptor co-agonists. Moreover, in individuals without clinical cardiorenal complications but with high CV risk, AD1 should be considered primary cardiorenal prevention; if there is a very high ASCVD risk, AD1 agents are recommended. If obesity is present, agents with efficacy in weight management should be considered, and GLP-1 RA should be the choice if high or very high CV risk is also present.

To avoid clinical inertia, the best strategy for naïve patients and treatment intensification in patients who have not achieved the HbA1c target was updated. Beyond dual therapy, triple therapy may also be considered when the initial HbA1c is between 7.5 and 9%. Moreover, triple therapy should be more consistently considered in asymptomatic adults with initial HbA1c above 9%. If insulin-based treatment (IBT) is indicated for a patient no longer using GLP-1 RA, a fixed-ratio co-formulation (FRC) insulin/GLP-1 RA should be considered over basal insulin or basal-bolus alone, whenever available. If obesity is present, the combination of basal insulin and GLP-1 RA titrated to the highest doses approved for weight loss should be considered. The periodicity of the HbA1c target evaluation was also updated, considering clinical aspects and cost–benefit issues.

In patients with established atherosclerotic cardiovascular disease (ASCVD), the 2023 UPDATE recommends SGLT2 inhibitors (SGLT2i) or GLP-1 RA as initial therapy. Metformin in association or a combination of GLP-1 RA and SGLT2i may also be considered to intensify blood glucose control. In patients with heart failure (HF), SGLT2i are now preferred independently of the ejection fraction, and intensification should be considered with metformin or GLP-1 RA. A warning for avoiding GLP-1 RA in patients with advanced HF with reduced ejection fraction was added due to the recent evidence of increased risk of ventricular arrhythmias in this scenario.

The algorithm for management of patients with T2D and renal disease was restructured, and estimated glomerular filtration rate (eGFR) plus albuminuria are critical references necessary for decisions. Although SGLT2i should not be initiated when eGFR is below 30 mL/min/1.73 m^2^, they can be maintained until dialysis.

## Methods

The main objective of this guideline was to support the decision-making process in clinical practice, considering the best evidence available. The panel was formed by 33 experts with extensive expertise in diabetes from both countries. Clinical topics requiring updated positions were ASCVD, HF, chronic kidney disease (CKD), and the management strategy for T2D in patients without vascular complications, focusing on controlling hyperglycemia and cardiorenal protection.

The panel compiled a narrative review by searching MEDLINE (via PubMed) for randomized clinical trials (RCTs), meta-analyses, and high-quality observational studies related to T2D. The best evidence available was reviewed, and when high-quality evidence was not available from the literature, the panel gave opinions on various clinical scenarios. These opinions were gathered and analyzed by an international voting system, allowing a consensus to be reached after multiple rounds of discussion.

**Fig. 1:**
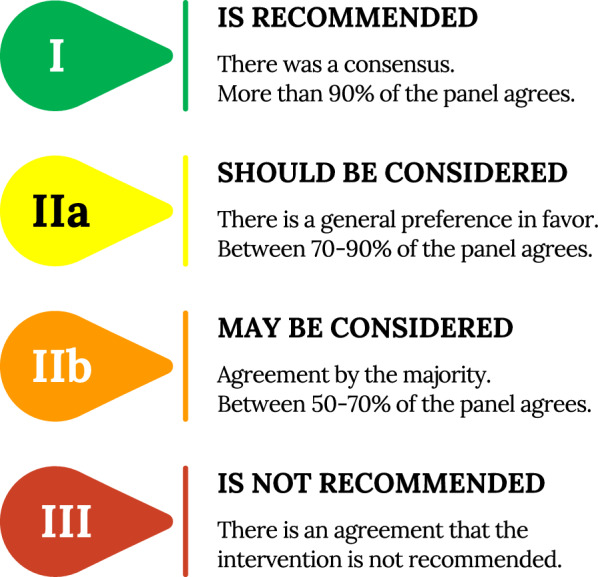
Class of recommendation

**Fig. 2:**
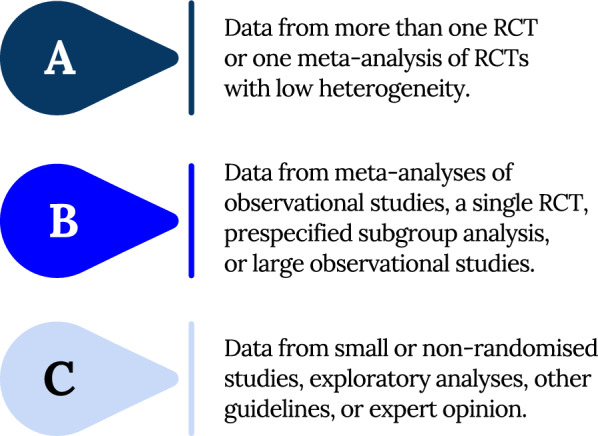
Level of evidence

A list of 45 statements was carefully created and scored according to the class of recommendation and level of evidence (Figs. 1 and 2).

## Recommendations

## General assessment

### R1

It IS RECOMMENDED that all treatment naïve adults with T2D have their cardiovascular risk status stratified, the renal function assessed, and body mass index, as well as HbA1c, determined before defining the use of antidiabetic agents.
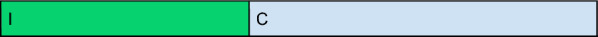


### Summary of evidence

**Fig. 3:**
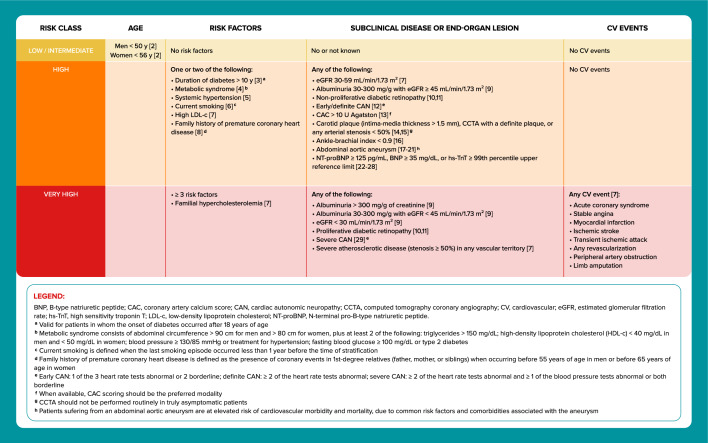
CV risk assessment in adults with T2D

- This panel considered assessing the cardiovascular (CV) risk essential to define the most appropriate antidiabetic treatment (Fig. 3). In general, the risk of long-term occurrence of CV events is twice as high in T2D compared to the general population of the same age [30]. The differences between individuals, however, are very heterogeneous according to age, the presence of risk factors, previous CV disease, previous CV events, and baseline renal function [1, 2, 9].
- The Emerging Risk Factors Collaboration group performed a meta-analysis of individual data from 102 prospective studies of patients with T2D without baseline cardiovascular disease [30]. Regressions were adjusted for age, sex, smoking, systolic blood pressure, and body mass index (BMI) to calculate vascular disease hazard ratios (HRs). The analysis included data from 698,782 people. Adjusted HRs with diabetes were: 2.00 (95% CI] 1.83 to 2.19) for coronary heart disease; 2.27 (95% CI 1.95 to 2.65) for ischemic stroke; 1.56 (95% CI 1.19 to 2.05) for hemorrhagic stroke; 1.84 (95% CI 1.59 to 2.13) for unclassified stroke and 1.73 (95% CI 1.51 to 1.98) for the combination of other vascular deaths. Overall, T2D conferred a twofold excess risk for a wide range of vascular diseases, independently from other risk factors.

## Glycemic targets

### R2

In adults with T2D, an HbA1c target of less than 7% IS RECOMMENDED to reduce the incidence of microvascular complications.
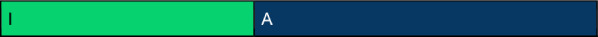


### Summary of evidence

- Improved blood-glucose control decreases the progression of diabetic microvascular disease. The UKPDS 33 trial [31] showed that reducing HbA1c to a target of less than 7% reduces microvascular complications. A total of 3867 newly diagnosed patients with T2D were randomly assigned to intensive treatment (sulfonylurea or insulin-based therapy [IBT]) or conventional treatment (diet alone). The intensive group aimed to attain fasting plasma glucose (FPG) of less than 108 mg/dL vs. the best achievable FPG with diet alone in the conventional group. Three aggregate endpoints were considered: (1) any diabetes-related endpoint (sudden death, death from hyperglycemia or hypoglycemia, fatal or non-fatal myocardial infarction [MI], angina, HF, stroke, renal failure, any amputation, vitreous hemorrhage, retinopathy requiring photocoagulation, blindness, or cataract extraction); (2) diabetes-related death (death from MI, stroke, peripheral vascular disease, renal disease, hyperglycemia or hypoglycemia, and sudden death); and (3) all-cause mortality (ACM). After ten years, the median HbA1c was 7% (interquartile range 6.2 to 8.2%) in the intensive group vs. 7.9% (6.9 to 8.8%) in the conventional group. For any diabetes-related endpoint, the risk was 12% lower in the intensive group (95% CI 1 to 21, P = 0.029) than in the conventional group. The risk reduction in any diabetes-related composite endpoint was attributable to a 25% risk reduction (95% CI 7 to 40, P = 0.0099) in microvascular outcome events.
- The frequency and severity of diabetic microvascular complications were examined in the Kumamoto study [32], a small randomized clinical trial (RCT) of 110 individuals with T2D observed for eight years. The study was divided into primary and secondary arms according to the presence of retinopathy to evaluate if intensive glycemic control could decrease the frequency or severity of microvascular complications. Patients were assigned to multiple insulin injections (MIT), administering three or more daily insulin injection therapy or conventional insulin injection therapy (CIT), administering 1 or 2 daily intermediate-acting insulin injections. In both primary and secondary prevention cohorts, the worsening in retinopathy and nephropathy were significantly lower (P < 0.05) in the MIT group than in the CIT group.

#### R3

In most adults with T2D, an HbA1c target of less than 7% IS RECOMMENDED to reduce the long-term incidence of macrovascular complications.
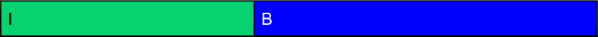


### Summary of evidence

- After UKPDS was finished, the post-trial observational phase monitored 3277 patients for five years, with no attempts to maintain their previously assigned therapies [33]. All patients were assessed through questionnaires, and seven prespecified aggregate clinical outcomes from the UKPDS were considered. Although between-group differences in HbA1c levels were lost after the first year, relative risk reductions persisted at ten years for any diabetes-related endpoint (9%, P = 0.04) and microvascular disease (24%, P = 0.001). A risk reduction for myocardial infarction (MI) (15%, P = 0.01) and all-cause mortality (ACM) (13%, P = 0.007) was observed. In the metformin group, significant risk reductions persisted for any diabetes-related endpoint (21%, P = 0.01), MI (33%, P = 0.005), and ACM (27%, P = 0.002). Despite an early loss of glycemic differences, a continued reduction in microvascular risk and risk reductions for MI and ACM was observed during the ten years of post-trial follow-up.
- The UKPDS 88 [34], a long-term observational follow-up from the original UKPDS study, examined the impact of early and delayed glucose-lowering therapy and the incidence of ACM and MI in T2D 20 years after randomization. The effect of HbA1c values over time was analyzed by weighting them according to their influence on following ACM and MI risks. HRs for a 1% higher HbA1c for ACM were 1.08 (95% CI 1.07 to 1.09), 1.18 (95% CI 1.15 to 1.21), and 1.36 (95% CI 1.30 to 1.42) at 5, 10, and 20 years, respectively for MI, was 1.13 (95% CI 1.11 to 1.15) at five years, increasing to 1.31 (95% CI 1.25 to 1.36) at 20 years. A 1% lower HbA1c from diagnosis generated an 18.8% (95% CI 21.1 to 16.0) ACM risk reduction 10–15 years later, whereas delaying this reduction until ten years after diagnosis showed a seven-fold lower 2.7% (95% CI – 3.1 to – 2.3) risk reduction. Early detection of diabetes and intensive glucose control from diagnosis is essential to decrease the long-term risk of glycemic complications.

#### R4

In frail older adults with T2D, a less strict HbA1c target, up to 8%, IS RECOMMENDED to minimize hypoglycemia without increasing mortality.
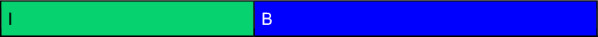


### Summary of evidence

- Glycemic targets must be individualized based on peoples' personal characteristics, needs, and preferences. In frail older adults with T2D, a less strict HbA1c target is recommended to minimize hypoglycemia. This panel highlights, however, that HbA1c should not exceed 8%, to avoid symptomatic hyperglycemia and increases in mortality in older adults with diabetes.
- An epidemiological study using the data from the NHANES III (1994–1998) of 7333 adults over 65 years analyzed mortality and the relationship between HbA1c and the risk of ACM and cause-specific mortality [35]. Compared with those with diagnosed diabetes and an HbA1c < 6.5%, the HR for ACM was significantly greater for adults with diabetes with an HbA1c > 8%. HRs were 1.6 (95% CI 1.02 to 2.6) and 1.8 (95% CI 1.3 to 2.6) for HbA1c 8–8.9% and ≥ 9%, respectively (P for trend < 0.001).
- In a retrospective cohort study from the Kaiser Permanente Northern California database [36], including 71,092 patients with T2D aging more than 60 years, the relationships between baseline HbA1c and subsequent outcomes (nonfatal complications [acute metabolic, microvascular, and CV events] and mortality) were analyzed. The mean cohort age was 71.0 ± 7.4 years, and the mean HbA1c was 7 ± 1.2%. The risk of any nonfatal complication rose when HbA1c ≥ 6% (adjusted HR 1.09, 95% CI 1.02 to 1.16, for HbA1c 6–6.9% and 1.86, 95% CI 1.63 to 2.13, for HbA1c ≥ 11%). Mortality, however, had a U-shaped relationship with HbA1c. Compared with HbA1c < 6%, mortality risk was lower when HbA1c was between 6–9% (e.g., 0.83, 95% CI 0.76 to 0.90, for HbA1c 7–7.9%) and higher when HbA1c ≥ 11% (1.31, 95% CI 1.09 to 1.57). The risk of any endpoint (complication or death) became significantly higher at HbA1c ≥ 8%. Patterns generally were consistent across age groups (60–69, 70–79, and ≥ 80 years).
- To investigate the association between HbA1c variability over time and mortality in older people with T2D, a 5-year retrospective cohort was assessed using The Health Improvement Network database [37]. The cohort included 587,000 primary care practices in the UK with patients of either sex who were above 70 years and older with type 1 or type 2 diabetes. The primary outcome was time to ACM. The primary exposure variables were mean HbA1c and variability of HbA1c over time. The observation included a 4-year run-in period with a 5-year follow-up from 2007 to 2012. A total of 54,803 people were enrolled, of whom 17,680 (8614 [30.7%] of 28,017 women and 9066 [33.8%] of 26,786 men) died during the observation period. The data showed a J-shaped distribution for mortality risk in both sexes, with significant increases in HbA1c values greater than 8% and less than 6%. Excess mortality risk was not significant for men at HbA1c values of 8% to less than 8.5%. Mortality increased with increasing HbA1c variability in all models (overall and for both sexes). Both low and high levels of glycemic control were associated with an increased mortality risk. The degree of variability also seems to be an essential factor, suggesting that a stable glycemic level in the middle range is associated with lower risk, and glycemic variability over time in HbA1c is essential in understanding mortality risk in older people with diabetes.

#### R5

It IS RECOMMENDED to measure HbA1c once every 12 weeks in patients that have not achieved the HbA1c target, after changing therapy, or in unstable situations.
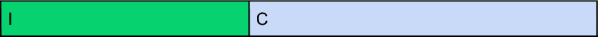


#### R6

It IS RECOMMENDED to measure HbA1c at least once every 24 weeks in patients meeting treatment goals.
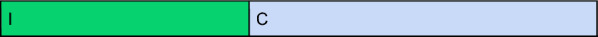


### Summary of evidence

- Recommendations 5 and 6 were based on the expert opinion of this panel, based on the current best clinical practice of Brazilian and Portuguese board members, considering cost-effective issues.

## Management of antidiabetic therapy in adults without cardiorenal disease

**Fig. 4:**
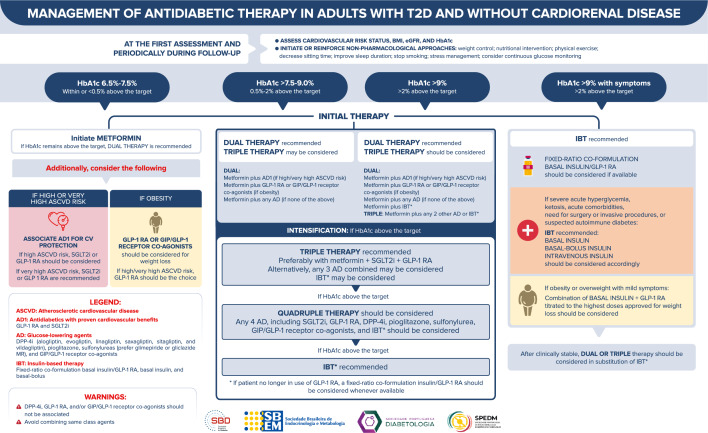
Management of antidiabetic therapy in adults with T2D and without cardiorenal disease

Figure 4 depicts the approach to managing antidiabetic therapy in adults with T2D and without cardiorenal disease.

### R7

Non-pharmacological approaches, such as nutritional intervention focusing on weight control, physical exercise, decreasing sitting time, improving sleep duration, stopping smoking, and stress management, ARE RECOMMENDED during all phases of treatment in T2D to improve glycemic control.
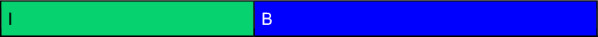


### Summary of evidence

- Lifestyle measures should be recommended universally as the basis for diabetes treatment, as sustained remission of T2D is related to the degree of weight loss.
- Weight loss is associated with sustained remission of T2D. The DIRECT study [38] was an open-label, cluster-randomized, controlled trial conducted at primary healthcare units in the United Kingdom (UK) that assessed remission of T2D during a direct care-led weight-management program. The study randomized overweight/obese patients recently diagnosed with T2D to an integrated structured weight management program (intervention) (n = 149) or the standard of care by UK guidelines (n = 149). The intervention included the withdrawal of antidiabetic drugs, total diet replacement (825–853 kcal/d formula diet for 12–20 weeks), and stepped food reintroduction (2–8 weeks), followed by structured support for weight-loss maintenance. The primary outcome was a weight loss of at least 15 kg and remission of T2D, defined as an HbA1c < 6.5% after withdrawal of antidiabetic agents at 12 and 24 months. At 24 months, 11% of patients in the intervention group and 2% of controls had achieved weight loss of at least 15 kg (odds ratio [OR] 7.49, 95% CI 2.05 to 7.32, P = 0.0023), and remission of diabetes was seen in 36% in the intervention group and 3% in the control group (OR 25.82, 95% CI 8.25 to 80.84, P < 0.0001). In a post hoc analysis of the whole study population, of those participants who maintained at least 10 kg weight loss (45 of 272 with data), 29 (64%) achieved remission; 36 (24%) of 149 participants in the intervention group maintained at least 10 kg weight loss.
- The association of sleep duration with CVD incidence and mortality in high-risk T2D populations was evaluated in a prospective study, which included 18,876 participants with T2D in the UK Biobank who were free of CVD and cancer at baseline [39]. During an average follow-up of 11–12 years, there were 2570 incident cases of ASCVD and 598 CVD deaths. Compared with sleeping for seven hours daily, the multivariable-adjusted HRs of ≤ 5 and ≥ ten h/d were 1.26 (95% CI 1.08 to 1.48) and 1.41 (95% CI 1.16 to 1.70) for incident ASCVD, 1.22 (95% CI 0.99 to 1.50) and 1.16 (95% CI 0.88 to 1.52) for coronary artery disease, 1.70 (95% CI 1.23 to 2.35) and 2.08 (95% CI 1.44 to 3.01) for ischemic stroke, 1.02 (95% CI 0.72 to 1.44) and 1.45 (95% CI 1.01 to 2.10) for peripheral artery disease, and 1.42 (95% CI 1.02 to 1.97) and 1.85 (95% CI 1.30 to 2.64) for CVD mortality. Short and long sleep durations were independently associated with increased risks of CVD onset and death among people with T2D.
- A meta-analysis [40] examined the association of total daily sitting time with CVD and T2D, with and without adjustment for physical activity. Nine studies with 448,285 participants were included. A higher real daily sitting time was associated with an increased risk of CVD (HR 1.29, 95% CI 1.27 to 1.30, P < 0.001) and T2D (HR 1.13, 95% CI 1.04 to 1.22, P < 0.001). The increased risk for T2D was not affected after adjusting for physical activity (HR 1.11, 95% CI 1.01 to 1.19, P < 0.001). The increased risk was attenuated for CVD but significant (HR 1.14, 95% CI 1.04 to 1.23, P < 0.001). The authors concluded that higher levels of total daily sitting time are associated with an increased risk of CVD and T2D, independent of physical activity. Therefore, the total daily sitting reduction is recommended in public health guidelines.
- A meta-analysis [41] of 47 studies assessing sedentary behavior in adults, adjusted for physical activity, was performed on outcomes for CVD and diabetes, cancer, and ACM. Inactive times were quantified using self-report. Significant HRs were found with ACM (HR 1.24, 95% CI 1.09 to 1.41), CVD mortality (HR 1.17, 95% CI 1.10 to 1.25), CVD incidence (HR 1.14, 95% CI 1.00 to 1.72), cancer mortality (HR 1.17, 95% CI 1.10 to 1.24), cancer incidence (HR 1.13, 95% CI 1.05 to 1.21), and T2D incidence (HR 1.91, 95% CI 1.64 to 2.22). HRs associated with sedentary time and outcomes were more pronounced at lower physical activity levels than higher ones. There was marked heterogeneity in research designs and the assessment of sedentary time and physical activity. Prolonged sedentary time was independently associated with deleterious health outcomes regardless of physical activity.

#### R8

Continuous glucose monitoring SHOULD BE CONSIDERED to improve glycemic control in T2D, taking into account the cost–benefit ratio.
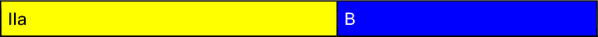


### Summary of evidence

- In a meta-analysis [42] of 13 real-world observational trials (data from 2415 participants) involving adults with T2D, the use of intermittently scanned continuous glucose monitoring (isCGM) was associated with a significant reduction in HbA1c. The fall in HbA1c occurred at 3–4 months (−0.45%, 95% CI – 0.57% to – 0.33%), continuing through 4.5–7.5 months (– 0.59%, 95% CI – 0.80% to – 0.39%) and was sustained after that for at least 12 months. The sustained reduction in HbA1c indicates that it is a consequence of using the isCGM system rather than transient confounding factors around initiation. Furthermore, meta-regression analysis shows that the degree of change in HbA1c was predicted by the HbA1c at baseline, such that a more significant reduction in HbA1c was seen for users with a higher baseline HbA1c.
- In a multicentric RCT [43] to determine the effectiveness of CGM in adults with T2D (n = 175) treated with basal insulin (without prandial insulin) in primary care practices, CGM resulted in significantly better glycemic control at eight months as compared with blood glucose meter (BGM) monitoring. Mean HbA1c level decreased from 9.1% at baseline to 8% at eight months in the CGM group and from 9% to 8.4% in the BGM group (adjusted difference – 0.4%, 95% CI – 0.8% to – 0.1%, P = 0.02). In addition, the mean percentage of CGM-measured time in the target glucose range of 70 to 180 mg/dL was 59% in the CGM group vs. 43% in the BGM group (adjusted difference 15%, 95% CI 8% to 23%, P < 0.001) and the mean percentage of time at greater than 250 mg/dL was 11% vs. 27%, respectively (adjusted difference – 16%, 95% CI – 21% to – 11%, P < 0.001). The mean glucose values were 179 mg/dL in the CGM group vs. 206 mg/dL in the BGM group (adjusted difference – 26 mg/dL, 95% CI – 41 to – 12, P < 0.001).
- The IMMEDIATE study [44] was a multisite, open-label, 16-week RCT to examine the efficacy and patient satisfaction of isCGM in non-insulin-treated adults with T2D. The participants (n = 116) were randomized 1:1 to receive a diabetes self-management education (DSME) plus isCGM (the isCGM + DSME group) or DSME plus blinded CGM (the DSME group). At 16 weeks of follow-up, the isCGM + DSME group had a significantly greater mean time in range (+ 9.9% [+ 2.4 h], P < 0.01), significantly less time above range (– 8.1% [– 1.9 h], P = 0.037), and a greater reduction in mean HbA1c (– 0.3%, 95% CI – 0.7% to 0%, P = 0.048) vs. the DSME group. The time below range was low and not significantly different between groups, and hypoglycemic events were few in both groups. Glucose monitoring satisfaction was higher among isCGM users (adjusted difference – 0.5, 95% CI – 0.7 to – 0.3, P < 0.01).

#### R9

In treatment-naïve adults recently diagnosed with T2D, without CVD or CKD, at low or intermediate CV risk, in whom HbA1c is 6.5–7.5%, metformin IS RECOMMENDED to improve glycemic control, mitigate diabetes progression, and prevent diabetes-related outcomes.
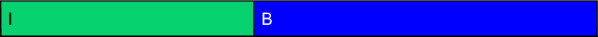


### Summary of evidence

- This panel concluded that, in T2D, metformin is highly efficacious in reducing hyperglycemia, well-tolerated, cheap, and safe, and can slow down the natural progression of T2D while reducing diabetes-related outcomes. However, the role of metformin in reducing CV outcomes is unclear.
- The UKPDS 34 study [45] investigated whether intensive blood-glucose control with metformin could reduce diabetes-related outcomes. In an RCT including 4075 participants, a subgroup of 1704 overweight people with newly diagnosed T2D was assigned to either conventional treatment with diet alone (n = 411), intensive control with metformin (n = 342), or intensive control with a sulfonylurea or IBT (n = 951). The median duration was 10.7 years. The primary outcome measures were any diabetes-related clinical endpoint, diabetes-related death, and ACM. The overall mean HbA1c at baseline was 7.2 ± 1.5%. Compared with the conventional group, patients in the metformin group had risk reductions of 32% (95% CI 13 to 47, P = 0.002) for any diabetes-related endpoint, 42% for diabetes-related death (95% CI 9 to 63, P = 0.017), and 36% for ACM (95% CI 9 to 55, P = 0.011). Among patients allocated to intensive glycemic control, metformin showed a more significant effect than chlorpropamide, glibenclamide (glyburide), or IBT for any diabetes-related endpoint (P = 0.0034), ACM (P = 0.021), and stroke (P = 0.032). Intensive glucose control with metformin decreased the risk of diabetes-related endpoints in overweight people with T2D. In addition, it was associated with less weight gain and fewer hypoglycemic attacks than IBT and sulfonylureas.
- Metformin can also mitigate the progression from prediabetes to T2D. The Diabetes Prevention Program (DPP) [46] was an RCT comparing intensive lifestyle intervention or metformin vs. placebo in a cohort of people with prediabetes who were selected at very high risk of developing T2D. After the trial, an observational phase, the DPP Outcome Study (DPPOS), which included 2776 (88%) of the surviving DPP cohort, was analyzed by intention-to-treat based on the original DPP assignment. During DPPOS, the lifestyle group was offered lifestyle reinforcement semi-annually, and the metformin group received unmasked metformin. During a mean 15 years of follow-up, lifestyle intervention and metformin reduced diabetes incidence rates by 27% (P < 0.0001) and 18% (P = 0.001), respectively, vs. the placebo group. There was an apparent decline in group differences over time. The cumulative incidences of T2D were 55%, 56%, and 62%, respectively, and the prevalence at the study-end of microvascular outcome composite outcome (nephropathy, neuropathy, and retinopathy) was not significantly different among the treatment groups (11–13%). Lifestyle intervention or metformin significantly reduced diabetes development over 15 years. There were no overall differences in the combined microvascular outcome among treatment groups. However, those who did not progress to diabetes had a lower prevalence of microvascular complications than those who progressed.

#### R10

In adults with T2D at high or very high CV risk, an AD1 IS RECOMMENDED for reduction of CV events.
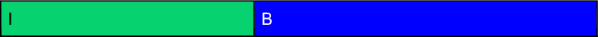


### Summary of evidence

- This panel defined as AD1 the anti-hyperglycemic agents with proven CV benefits, i.e., SGLT2 inhibitors (SGLT2i) and glucagon-like peptide-1 receptor agonists (GLP-1 RA).
- SGLT2i favorably affects CV events and CV mortality in high-risk adults with T2D. A meta-analysis [47] of 6 randomized, placebo-controlled CV outcomes trials (CVOTs) with SGLT2i included data from 6 trials comprising 46,969 patients with T2D, 66.2% with ASCVD. Overall, SGLT2i reduced the risk of MACE by 10% (HR 0.90, 95% CI 0.85 to 0.95), with no significant heterogeneity of associations with outcome. The presence or absence of ASCVD did not modify the association with outcomes for MACE (P for interaction = 0.10). There was also no difference between the subgroups with baseline HbA1c above or below 8.5% (P for interaction = 0.09). SGLT2i also reduced CV mortality by 15% (HR 0.85, 95% CI 0.78 to 0.93, without differences between patients with or without previous ASCVD; P for interaction = 0.44). These data support recommendations to prioritize the use of SGLT2i in patients at high ASCVD risk.
- GLP-1 RA reduces MACE, CV mortality, and ACM in high-risk patients with T2D. In a meta-analysis [48] including data from 8 trials comprising 60,080 patients, GLP-1 RA reduced MACE by 14% (HR 0.86, 95% CI 0.80 to 0.93), with no significant heterogeneity between subgroups with or without established ASCVD (P for interaction = 0.18). Overall, GLP-1 RA reduced CV mortality by 13% (HR 0.87, 95% CI 0.80 to 0.94) and ACM by 12% (HR 0.88, 95% CI 0.82 to 0.94), with no increase in the risk of severe hypoglycemia, retinopathy, or pancreatic adverse effects. This data supports current recommendations to prioritize the use of GLP-1 RA in patients at high ASCVD risk.

#### R11

In adults with T2D and obesity, GLP-1 RA or GIP/GLP-1 receptor co-agonists SHOULD BE CONSIDERED for improving weight loss.
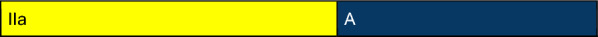


### Summary of evidence

- The STEP 2 study [49] was a double-blind, double-dummy, randomized phase 3 clinical trial that assessed the efficacy and safety of the once-a-week subcutaneous GLP-1 RA semaglutide, in doses of 2.4 mg vs. 1.0 mg vs. placebo, for weight management in adults with T2D and overweight or obesity. The study enrolled adults with a BMI ≥ 27 kg/m^2^ and HbA1c 7–10% who had been diagnosed with T2D for at least 180 days before screening. Patients were randomly allocated (1:1:1) via an interactive web-response system and stratified by background glucose-lowering medication and HbA1c to SC injection of semaglutide 2.4 mg, semaglutide 1.0 mg, or visually matching placebo, once a week for 68 weeks, plus a lifestyle intervention. Co-primary endpoints were percentage change in body weight and achievement of weight reduction of at least 5% at 68 weeks for semaglutide 2.4 mg vs. placebo, assessed by intention to treat. A total of 1210 were randomly assigned to semaglutide 2.4 mg (n = 404), semaglutide 1.0 mg (n = 403), or placebo (n = 403) and included in the intention-to-treat analysis. The estimated change in mean body weight from baseline to week 68 was – 9.6% with semaglutide 2.4 mg vs. – 3.4% with placebo. The estimated treatment difference (ETD) for semaglutide 2.4 mg vs. placebo was – 6.2% (95% CI – 7.3 to – 5.2; P < 0.0001). At week 68, more patients on semaglutide 2.4 mg than on placebo achieved weight reductions of at least 5% (267 [68.8%] of 388 vs. 107 [28.5%] of 376; OR 4.88, 95% CI 3.58 to 6.64, P < 0.0001). In adults with overweight/obesity and T2D, semaglutide 2.4 mg once a week significantly decreased body weight compared with placebo.
- The SURPASS 1 study [50] was a 40-week, double-blind, randomized, placebo-controlled, phase 3 trial to assess efficacy, safety, and tolerability of GIP/GLP-1 receptor co-agonist tirzepatide monotherapy vs. placebo in adults with T2D inadequately controlled by diet and exercise alone. The primary endpoint was the mean change in HbA1c from baseline at 40 weeks. A total of 478 individuals were randomly assigned to tirzepatide 5 mg (n = 121 [25%]), 10 mg (n = 121 [25%]), 15 mg (n = 121 [25%]), or placebo (n = 115 [24%]). At 40 weeks, all tirzepatide doses were superior to placebo for changes from baseline in HbA1c, fasting serum glucose, body weight, and HbA1c targets of < 7% and < 5.7%. Mean HbA1c decreased from baseline by 1.87% with tirzepatide 5 mg, 1.89% with tirzepatide 10 mg, and 2.07% with tirzepatide 15 mg vs. + 0.04% with placebo, resulting in estimated treatment differences vs. placebo of – 1.91%, – 1.93%, and – 2.11%, respectively (all P < 0.0001). More participants on tirzepatide than on placebo met HbA1c targets of < 7% (87–92% vs. 20%) and ≤ 6.5% (81–86% vs. 10%), and 31–52% of patients on tirzepatide vs. 1% on placebo reached an HbA1c < 5.7%. Tirzepatide induced a dose-dependent body weight loss ranging from 7 to 9.5 kg. Tirzepatide showed important improvements in glycemic control and body weight without increased risk of hypoglycemia. The safety profile was consistent with GLP-1 RA, indicating a potential monotherapy use of tirzepatide for T2D treatment.

#### R12

In treatment-naïve asymptomatic adults with T2D, at low or intermediate CV risk, in whom HbA1c is above 7.5%, dual therapy, including metformin and a second AD1 or AD, IS RECOMMENDED to improve glycemic control.
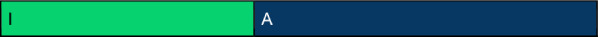


### Summary of evidence

Adding SGLT2 inhibitors:

- Compared with placebo, SGLT2i reduced HbA1c levels when used as monotherapy (weighted mean difference [WMD] 0.79%, 95% CI 0.96% to 0.62%, I^2^ 71%) or add-on treatment (WMD 0.61%, 95% CI 0.69% to 0.53%, I^2^ 73%) [51].

Adding GLP-1 Receptor Agonists:

- The efficacy of adding liraglutide to metformin was compared with the addition of placebo or glimepiride to metformin in subjects previously treated with oral antidiabetic therapy. In a 26-week, double-blind, double-dummy, placebo, and active-controlled, parallel-group trial, 1091 adults with T2D were randomly assigned to once-daily liraglutide (either 0.6, 1.2, or 1.8 mg/d injected SC), to placebo, or to glimepiride (4 mg once daily) [52]. All treatments were in combination therapy with metformin (1 g twice daily). Baseline HbA1c was 7–11% if on previous monotherapy > 3 months or 7–10% if previous dual therapy > 3 months. HbA1c values were reduced in all liraglutide groups vs. the placebo group (P < 0.0001), with mean decreases of 1% for 1.8 and 1.2 mg liraglutide and glimepiride and 0.7% for 0.6 mg liraglutide vs. an increase of 0.1% for placebo. Liraglutide induced similar glycemic control, reduced body weight, and lowered the occurrence of hypoglycemia compared with glimepiride, when both had background therapy with metformin.

Adding DPP-4 inhibitors:

- Dual therapy with DPP-4i and metformin is efficacious and safe. A meta-analysis [53] assessing the long-term efficacy and safety of DPP-4i combined with metformin compared to metformin alone in patients with T2D included seven RCTs lasting at least 24 weeks. The decline in HbA1c was greater with dual therapy. The difference was – 0.54% (95% CI – 0.63 to – 0.45), with no increase in hypoglycemia (HR 0.79, 95% CI 0.48 to 1.30).

Adding pioglitazone:

- The addition of pioglitazone (30 mg/d) to other antidiabetic agents (metformin or sulfonylureas) led to more significant reductions in HbA1c level by – 1.16% (95% CI – 1.41 to – 0.90) compared with placebo [54].

Adding sulfonylureas:

- The safety of sulfonylureas in relation to CV outcomes was demonstrated in the CAROLINA head-to-head RCT [55] (glimepiride vs. linagliptin) in the TOSCA.IT head-to-head trial [56] (glimepiride vs. pioglitazone), and in the ADVANCE trial [57] (gliclazide MR).
- In a meta-analysis [58] of RCTs, CV safety was also extended to glibenclamide (glyburide). This panel considered that sulfonylureas are safe in relation to CV risk. However, they are associated with an increased incidence of hypoglycemia. Therefore, prescriptions must be individualized for each patient.
- Among the sulfonylureas, gliclazide MR is associated with a lower risk of hypoglycemia. In the GUIDE trial [59], a head-to-head comparison of gliclazide MR and glimepiride (n = 845), hypoglycemia occurred less frequently with gliclazide MR than with glimepiride (3.7% vs. 8.9%, respectively; P = 0.003).

Adding GIP/GLP-1 receptor co-agonists:

- A systematic review and meta-analysis [60] evaluating the efficacy and safety of tirzepatide against placebo or active comparator in people with T2D included six RCT (data from 6579 subjects; 4410 in the tirzepatide group and 2054 in the control group). Tirzepatide treatment reduced HbA1c, the primary endpoint (WMD  – 1.07%, 95% CI – 1.44 to – 0.56, I^2^ 98%). Secondary efficacy endpoints also improved with tirzepatide. Fasting serum glucose (WMD – 21.50 mg/dL, 95% CI – 34.44 to – 8.56), body weight (WMD – 7.99 kg, 95% CI – 11.36 to – 4.62, I^2^ 99%), blood pressure, and fasting lipid profiles, without increasing hypoglycemia, either as monotherapy or add-on therapy. Tirzepatide increased the risk of gastrointestinal adverse events (risk ratio 3.32, 95% IC 1.3 to 8.5, I^2^ 95%) as add-on therapy, but not in terms of pancreatitis or cholelithiasis. Furthermore, tirzepatide presented a dose-response effect (1 mg to 15 mg) on decreased HbA1c and body weight.

#### R13

In treatment-naïve asymptomatic adults with T2D, in whom HbA1c is 7.5% to 9%, triple therapy, including metformin and two AD1 or AD, MAY BE CONSIDERED to improve glycemic control.
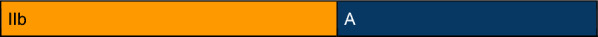


### Summary of evidence

- This panel considered that, in general, triple therapy is effective and safe for improving glycemic control. In addition, most studies indicate superior HbA1c-lowering efficacy with triple than with dual therapy. Therefore, it is likely that patients with HbA1c closer to 9% are potential candidates for initial triple therapy.
- Considering the combination of metformin, SGLT2i and GLP-1 RA, the AWARD-10 trial [61] randomized 424 patients who were on SGLT2i and metformin to receive dulaglutide 1.5 mg (n = 142), dulaglutide 0.75 mg (n = 142), or placebo (n = 140). The primary objective was to test for superiority of dulaglutide vs. placebo regarding the change in HbA1c from baseline at 24 weeks. HbA1c was reduced further in patients receiving all three drugs (dulaglutide 1.5 mg: – 1.34% ± 0.06 and dulaglutide 0.75 mg: – 1.21% ± 0.06) than in those receiving two drugs (placebo plus metformin/SGLT2i: – 0.54% ± 0.06, P < 0.0001). Triple therapy improved glycemic control significantly, with acceptable tolerability.
- The DURATION-8 study [62] was a 28-week, multicenter, double-blind, active-control trial of T2D patients with HbA1c 8–12% who were on metformin monotherapy. Patients (n = 695) were randomly assigned to receive exenatide plus dapagliflozin, exenatide plus placebo, or dapagliflozin plus placebo. The primary endpoint was a change in HbA1c from baseline to week 28. At 28 weeks, the change in HbA1c was – 2% (95% CI – 2.2 to – 1.8) in the exenatide/dapagliflozin group, – 1.6% (95% CI – 1.8 to – 1.4) in the exenatide group, and – 1.4% (95% CI – 1.6 to – 1.2) in the dapagliflozin group. The combination of exenatide and dapagliflozin significantly reduced HbA1c from baseline to week 28 compared with exenatide alone (– 0.4%, 95% CI – 0.6 to – 0.1, P = 0.003) or dapagliflozin alone (– 0.6%, 95% CI – 0.8 to – 0.3, P < 0.001), and was well tolerated.
- The combination of empagliflozin and linagliptin was examined as second-line therapy in subjects with T2D inadequately controlled on metformin in a double-blind RCT [63]. Patients were randomized to empagliflozin plus linagliptin or each drug alone in different dosages as an add-on to metformin for 52 weeks. The primary endpoint was the change in HbA1c from baseline at week 24. At week 24, decreases in HbA1c from a baseline of 7.90–8.02% were superior with empagliflozin/linagliptin than with empagliflozin 25 mg or linagliptin 5 mg alone as add-ons to metformin. Overall, 61.8% attained HbA1c < 7% with the combination of empagliflozin 25 mg/linagliptin 5 mg, while only 32.6% did with empagliflozin 25 mg alone (OR 4.2, 95% CI 2.3 to 7.6, P < 0.001), and 36.1% with linagliptin 5 mg alone (OR 3.5, 95% CI 1.9 to 6.4, P < 0.001). Efficacy was maintained at week 52. The proportion of subjects with adverse events over 52 weeks was similar across treatment arms (68.6–73%), with no hypoglycemic events requiring assistance.
- The empagliflozin/linagliptin combination as second-line therapy for 52 weeks significantly reduced HbA1c compared with the individual components and was well tolerated. In an open-label clinical trial [64], 106 patients recently diagnosed with T2D were randomized to metformin/pioglitazone/exenatide (triple therapy) and 115 to metformin, followed by sulfonylurea and glargine U100 (conventional treatment) with an HbA1c target of < 6.5% for two years. Patients receiving triple therapy had a more significant reduction in HbA1c level than those receiving conventional treatment (5.95% vs. 6.50%; P < 0.001). In addition, despite lower HbA1c, participants on triple therapy experienced a 7.5-fold lower rate of hypoglycemia than patients on conventional treatment. Triple therapy was also associated with weight loss vs. weight gain in those receiving conventional treatment (−1.2 kg vs. + 4.1 kg, respectively; P < 0.01).
- A post hoc analysis [65] of three RCTs of sequential or concomitant add-on of dapagliflozin and saxagliptin to metformin compared the safety of triple therapy (dapagliflozin plus saxagliptin + metformin) vs. dual therapy (dapagliflozin or saxagliptin plus metformin). At 24 weeks, the incidence of any adverse and serious adverse events was similar between the triple and dual therapy groups and between the concomitant and sequential add-on groups. Urinary tract infections were more common in the sequential groups than concurrent groups; genital infections were reported only with the sequential add-on of dapagliflozin to saxagliptin plus metformin. Hypoglycemia occurred in < 2% of patients across all groups.
- A network meta-analysis [66] compared the efficacy of adding a third AD in patients with T2D not well controlled (HbA1c > 7%) by dual therapy with metformin and sulfonylurea. The meta-analysis included only RCTs of at least 24 weeks’ duration. The primary outcomes were a change in HbA1c, weight change, and severe hypoglycemia frequency. A total of 18 trials involving 4,535 participants, with a mean duration of 31 weeks, were included. Compared with placebo, drug classes did not differ regarding the effect on HbA1c level, with reductions ranging from − 0.70% (95% CI – 1.33% to – 0.08%) to – 1.08% (95% CI – 1.41% to – 0.77%). Weight gain was seen with IBT (2.84 kg, 95% CI 1.76 to 3.90 kg) and with thiazolidinediones (4.25 kg, 95% CI 2.76 to 5.66 kg), while weight loss was seen with GLP-1 RA (– 1.63 kg, 95% CI – 2.71 to – 0.60 kg). IBT caused twice more severe hypoglycemic episodes than non-insulin ADs. No agent was superior to any other in terms of HbA1c.

#### R14

In treatment-naïve, asymptomatic adults with T2D, in whom HbA1c > 9%, metformin plus IBT SHOULD BE CONSIDERED to improve glycemic control.
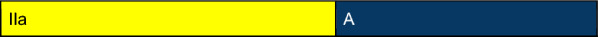


### Summary of evidence

- A meta-analysis [67] comparing CV and metabolic outcomes in insulin-based vs. non-insulin-based glucose-lowering therapy included 18 RCTs (data from 19,300 patients). In 16 trials, insulin had superior efficacy in achieving glycemic control (HR 0.20, 95% CI 0.28 to 0.11) and was associated with superior reductions in HbA1c. Baseline HbA1c among all included studies ranged from 7.4 to 9.7%. There was no significant between-group difference in ACM or CV events risk. However, the risk of hypoglycemia was higher among patients receiving insulin (relative risk 1.90, 95% CI 1.44 to 2.51). Non-insulin treatment was associated with more adverse drug reactions (54.7% vs. 45.3%, P = 0.044).
- Compared with oral ADs, early intensive insulin therapy in patients with newly diagnosed T2D is associated with a favorable impact on recovery and maintenance of β-cell function, as well as prolonged glycemic remission. A multicenter RCT [68] compared the effects of transient intensive insulin therapy (continuous subcutaneous insulin infusion [CSII] or multiple daily injections [MDI]) vs. oral antidiabetic agents on β-cell function and diabetes remission. A total of 382 treatment-naïve patients with recently diagnosed T2D were randomized to receive insulin or oral hypoglycemic agents for rapid initial correction of hyperglycemia. The mean HbA1c at baseline was 9.5–9.8%. Treatment was stopped once normoglycemia had been achieved and remained stable for two weeks; patients were then followed on a diet and exercise alone. Intravenous glucose tolerance tests were performed, and glucose, insulin, and proinsulin levels were measured. The primary endpoint was the duration of glycemic remission and remission rate at one year. More patients achieved target glycemic control in the insulin groups than those treated with oral ADs. In addition, the 1-year remission rate was significantly higher in the insulin groups (51.1% and 44.9% vs. 26.7% with oral ADs; P = 0.0012). β-cell function, assessed by the homeostasis model assessment of β-cell function (HOMA-β) and acute insulin response, also improved significantly after intensive therapy. The increase in acute insulin response was sustained in the insulin groups but considerably declined in the oral ADs group at one year in all patients who achieved remission.

#### R15

In treatment-naïve, asymptomatic adults with T2D, in whom HbA1c > 9%, triple therapy including metformin and two other AD1 or AD SHOULD BE CONSIDERED to improve glycemic control.
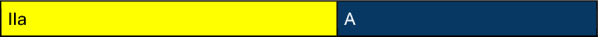


### Summary of evidence

- the summary of evidence in recommendation 13.

#### R16

In adults with T2D, HbA1c > 9%, and signs or symptoms of hyperglycemia (polyuria, polydipsia, weight loss), insulin-based therapy IS RECOMMENDED to improve glycemic control.
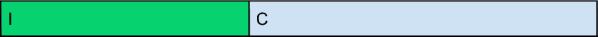


### Summary of evidence

- This panel recommended using insulin-based therapy (IBT) in T2D patients with symptoms of hyperglycemia. There is general agreement that IBT is necessary when signs or symptoms of insulin deficiency are present. This statement is based primarily on the pathophysiology of T2D, plausibility, and clinical experience.

#### R17

In adults with T2D, obesity, and HbA1c > 9%, without severe signs or symptoms of hyperglycemia, a combination of basal insulin and GLP-1 RA therapy SHOULD BE CONSIDERED to improve glycemic control.
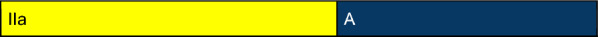


### Summary of evidence

- A meta-analysis of RCTs [69] assessed the efficacy and safety of short and long-acting GLP-1 RA, both used in combination with basal insulin, in adults with T2D. A total of 14 RCTs were included. Eight trials examined short-acting and six long-acting GLP-1 RA. Differences in HbA1c, fasting plasma glucose, body weight, and adverse events were compared between studies using short-or long-acting GLP-1 RA. Long-acting GLP-1 RA was more effective in reducing HbA1c (∆ -6 mmol/mol, 95% CI – 10 to – 2, P = 0.007), fasting plasma glucose (∆ – 0.7 mmol/L, 95% CI – 1.2 to – 0.3, P = 0.007), and body weight (∆ – 1.4 kg, 95% CI – 2.2 to – 0.6, P = 0.002) and raised the proportion of patients achieving an HbA1c target < 7% (P = 0.03) more than the short-acting ones. Furthermore, patients reporting symptomatic (P = 0.048) but not severe (P = 0.96) hypoglycemia were fewer with long- vs. short-acting GLP-1 RA added to insulin. In addition, a lower proportion of patients reported nausea (– 52%, P < 0.0001) or vomiting (– 36%, P = 0.0002) with long-acting GLP-1 RA. GLP-1 RA improved HbA1c, fasting plasma glucose, and body weight when added to basal insulin. Long-acting GLP-1 RA, however, was significantly more effective for glycemic and body weight control and displayed better gastrointestinal tolerability.

### Intensification of blood glucose control

#### R18

In adults with T2D and without cardiorenal complications, whose HbA1c remains above target despite dual therapy, triple therapy IS RECOMMENDED to improve glycemic control.
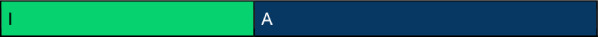


### Summary of evidence

- See the summary of evidence in recommendation 13.

#### R19

In adults with T2D without cardiovascular or renal complications, whose HbA1c remains above target despite triple therapy, quadruple therapy IS RECOMMENDED to improve glycemic control.
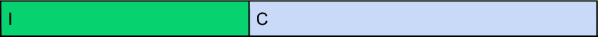


### Summary of evidence

- Quadruple therapy was evaluated in an open-label observational trial [70] in patients with uncontrolled T2D (HbA1c 7.5–12%) despite three oral ADs. The objective was to address the effectiveness and safety of adding empagliflozin or glargine U100 as a fourth agent in patients already on metformin, DPP-4i, and glimepiride. A total of 268 patients were included: 142 on empagliflozin (25 mg/d) and 126 on glargine U100. After 24 weeks, HbA1c reduced from baseline by 1.5 ± 1.2% (P < 0.001) in the empagliflozin group and by 1.1 ± 1.8% (P < 0.001) in the glargine U100 group. Moreover, HbA1c and FPG were significantly reduced (HbA1c, P = 0.004; FPG, P = 0.008, respectively) in the empagliflozin group vs. the glargine U100 group. In addition, hypoglycemic adverse events were significantly higher in the glargine U100 group vs. the empagliflozin group (P = 0.001). Therefore, quadruple therapy with SGLT2i, metformin, DPP-4i, and sulfonylurea was effective and safe for treating T2D.
- An open-label, prospective, 52-week study [71] was conducted in T2D to compare the effectiveness and safety of adding empagliflozin 25 mg/d or dapagliflozin ten mg/d as part of a quadruple therapy regimen for patients already on metformin, glimepiride, and DPP-4i, and still inadequately controlled (HbA1c 7.5–12%). The primary outcome was a change in HbA1c. In total, 350 patients were enrolled to receive empagliflozin (n = 176) or dapagliflozin (n = 174). After 52 weeks, both groups had significant reductions in HbA1c. The decline, however, was more important in the empagliflozin group (P < 0.001). Safety profiles were similar in the two groups, demonstrating that quadruple therapy can be used effectively in patients with T2D.

#### R20

In adults with T2D whose HbA1c remains above target despite quadruple therapy, adding insulin-based therapy IS RECOMMENDED to improve glycemic control.
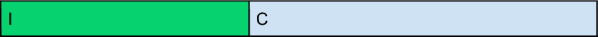


### Summary of evidence

- In a 26-week open-label trial [72], patients receiving GLP-1 RA therapy (liraglutide once daily or exenatide twice daily) plus metformin alone or metformin plus pioglitazone and a sulfonylurea were randomly assigned to receive insulin degludec plus liraglutide once daily (n = 292) or to continue GLP-1 RA therapy and oral ADs at the pre-trial dose (n = 146). At 26 weeks, superior HbA1c reductions had been achieved with the insulin degludec/liraglutide combination (ETD – 0.94%, P < 0.001).

#### R21

In asymptomatic adults with T2D requiring IBT, a fixed-ratio co-formulation insulin/GLP-1 RA SHOULD BE CONSIDERED over basal insulin or basal-bolus insulin, whenever available, to improve glucose control.
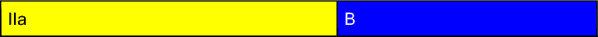


### Summary of evidence

- A preplanned subgroup analysis of a meta-analysis [73] included 6 RCTs (n = 4,213) comparing fixed-ratio co-formulation (FRC) insulin/GLP-1 RA vs. up-titration of basal insulin on metabolic control in adults with T2D. All trials had at least 24 weeks’ duration of intervention, and, for the most, the control group was on glargine U100 or degludec. The FRC therapy led to a mean HbA1c decrease significantly greater than basal insulin up-titration (WMD – 0.50%, 95% CI – 0.67 to – 0.33%, P < 0.001, I^2^ 91%), more patients at HbA1c target (relative risk [RR] 1.48, 95% CI 1.23 to 1.77, P < 0.001, I^2^ 92.3%), similar hypoglycemic events (RR 0.87, 95% CI 0.72 to 1.04, P = 0.114, I^2^ 72.9%), and weight reduction (WMD – 2.0, 95% CI – 2.6 to – 1.4, P < 0.001, I^2^ 86%).
- A RCT [74] assessed the efficacy and safety of initiating FRC insulin degludec/liraglutide vs. basal-bolus insulin in adults with uncontrolled T2D under basal insulin and metformin. All participants were randomized to FRC or glargine U100 plus insulin aspart up to 4 times daily. The FRC elicited HbA1c reductions comparable to basal-bolus (ETD 0.02%, 95% CI – 0.16 to 0.12); non-inferiority confirmed (P < 0.0001). The number of severe or confirmed symptomatic hypoglycemia events was lower with co-formulation vs. basal-bolus (risk ratio 0.39, 95% CI 0.29 to 0.51), and body weight decreased with co-formulation and increased with basal-bolus (ETD 23.6 kg, 95% CI 24.2 to 22.9). Total daily insulin dose was lower with co-formulation (40 units) than basal-bolus (40 units vs. 84 units total [52 units basal], respectively; ETD – 44.5 units, 95% CI 248.3 to 240.7, P < 0.0001). By week 26, approximately 90% of patients on basal-bolus reported taking at least three insulin injections per day vs. the once-daily single injection with FRC.
- A retrospective analysis of an extensive database [75] compared outcomes in adults with T2D under basal insulin therapy who were newly initiated on FRC insulin glargine U100/lixisenatide or basal-bolus insulin therapy. Cohorts were propensity score–matched in a 1:1 ratio on baseline characteristics (n = 2,140; 1,070 individuals in each group). The primary endpoint was persistence with therapy at 12 months. Secondary endpoints included treatment adherence, hypoglycemia, and HbA1c change at 12 months. Treatment persistence was higher for FRC vs. basal-bolus (HR 0.51, 95% CI 0.46 to 0.57, adjusted P < 0.001). In addition, adherence was higher (adjusted OR 4.00, 95% CI 3.25 to 4.91) and hypoglycemic events were lower (adjusted RR 0.61, 95% CI 0.45 to 0.84) for FRC vs. basal-bolus. HbA1c reduction from baseline, however, was slightly more significant for basal-bolus insulin therapy (0.65 vs. 0.84%, least squares mean [LSM] 0.58 vs. 0.73%, LSM difference 0.15%, 95% CI 0.04 to 0.34).

## Management of antidiabetic therapy in adults with T2D and atherosclerotic cardiovascular disease (ASCVD)

**Fig. 5:**
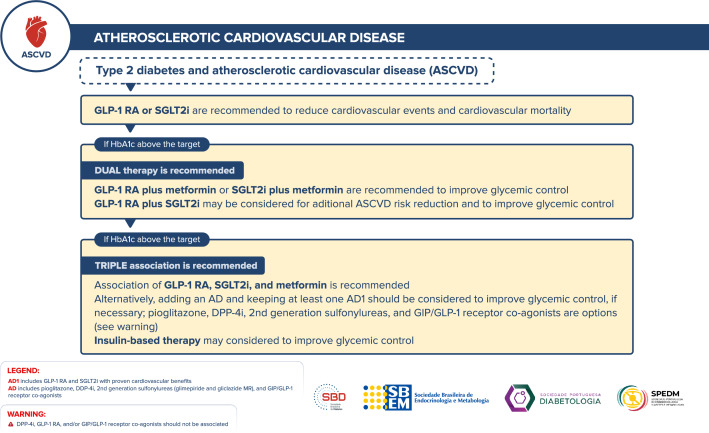
Management of antidiabetic therapy in adults with T2D and ASCVD

Figure 5 depicts the approach to managing antidiabetic therapy in adults with T2D and ASCVD.

### R22

In adults with T2D with clinical ASCVD, SGLT2i or GLP-1 RA (AD1) ARE RECOMMENDED to reduce cardiovascular events and CV mortality.
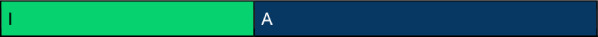


### Summary of evidence

- SGLT2i favorably affects CV events and CV mortality in high-risk adults with T2D. A meta-analysis [47] included data from 6 CVOTs of SGLT2i, comprising 46,969 unique patients with T2D and 31,116 (66.2%) with ASCVD. The primary outcomes were MACE and each one of its components (MI, stroke, or CV death). Overall, SGLT2i reduced the risk of MACE by 10% (HR 0.90, 95% CI 0.85 to 0.95), with no significant heterogeneity of associations with outcome. The presence or absence of ASCVD did not modify the association with outcomes for MACE (P for interaction = 0.10). Specifically, in patients with ASCVD, the HR was 0.89 (95% CI 0.84 to 0.95). There was also no difference between the subgroups with baseline HbA1c below or above 8.5% (P for interaction = 0.09). SGLT2i also reduced CV mortality by 15% (HR 0.85, 95% CI 0.78 to 0.93), without differences between patients with or without previous ASCVD (P for interaction = 0.44). Specifically, in patients with ASCVD, the HR was 0.83 (95% CI 0.76 to 0.92).
- GLP-1 RA reduces MACE, CV mortality, and ACM in high-risk patients with T2D. In a meta-analysis [48] including eight trials, comprising data from 60,080 patients, GLP-1 RA reduced MACE by 14% (HR 0.86, 95% CI 0.80 to 0.93), with no significant heterogeneity between patients with or without ASCVD (P for interaction = 0.94) or HbA1c baseline values (P for interaction = 0.14). Specifically, in patients with ASCVD, the HR was 0.85 (95% CI 0.78 to 0.92). Overall, GLP-1 RA also reduced CV mortality by 13% (HR 0.87, 95% CI 0.80 to 0.94) and ACM by 12% (HR 0.88, 95% CI 0.82 to 0.94).
- In a meta-analysis [76] of 6 RCTs with SGLT2i (data from 51,743 participants), CV outcomes and mortality were stratified according to baseline metformin use, ranging from 21 to 82%. SGLT2i reduced the risk of MACE, with and without concomitant metformin use (HR 0.93, 95% CI 0.87 to 1.00 and HR 0.82, 95% CI 0.71 to 0.86, respectively; P for interaction = 0.14). Treatment with SGLT2i results in clear and consistent reductions in CV outcomes and mortality regardless of whether patients are receiving or not receiving metformin.
- Despite the lower risk of CV events in patients treated with canagliflozin [77] or injectable semaglutide [78] vs. placebo, it is essential to note that, in the CANVAS Program [77], patients treated with canagliflozin had a greater risk of amputation (HR 1.97, 95% CI 1.41 to 2.75), primarily at the level of the toe or metatarsal; in the SUSTAIN-6 trial [78], rates of retinopathy complications (vitreous hemorrhage, blindness, or conditions requiring treatment with an intravitreal agent or photocoagulation) were significantly higher (HR 1.76, 95% CI 1.11 to 2.78, P = 0.02) in those who received injectable semaglutide. These adverse effects are new findings for which the mechanisms are unknown. Therefore, this panel recommended caution in using canagliflozin in patients at risk for amputation and injectable semaglutide in those with proliferative retinopathy.

#### R23

In adults with T2D and clinical ASCVD, who are in use of either SGLT2i or a GLP-1 RA, combining GLP-1 RA plus SGLT2i MAY BE CONSIDERED, as it is associated with fewer CV events and decreased all-cause mortality.
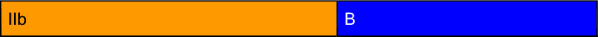


### Summary of evidence

- In a large, real-world observational study [79], 12,584 adults with T2D that received either SGLT2i or sulfonylureas to baseline GLP-1 RA were identified within 3 United States datasets. Subjects were 1:1 matched, using the propensity score, adjusting for baseline covariates. The composite CV endpoint included MI, stroke, and ACM. The adjusted pooled HR of SGLT2i initiators vs. sulfonylureas initiators was 0.76 (95% CI 0.59 to 0.98). This decrease in the primary outcome was driven by reductions in the risk of MI (HR 0.71, 95% CI 0.51 to 1.003) and ACM (HR 0.68, 95% CI 0.40 to 1.14) but not stroke (HR 1.05, 95% CI 0.62 to 1.79). In this cohort already on GLP-1 RA, the association with SGLT2i vs. sulfonylurea was associated with a more significant CV benefit.
- In an exploratory analysis of the AMPLITUDE-O trial [80], the effects of the GLP-1 RA efpeglenatide on MACE, expanded MACE, renal composite outcome, MACE, or death outcome, and hospitalizations for heart failure (hHF), as well as adverse events, appeared to be independent of concurrent SGLT2i use, as judged by point estimates in patients receiving compared with those not receiving baseline SGLT2i and lack of any formal interactions. These data support combined SGLT2i and GLP-1 RA therapy in T2D.
- To evaluate the effects of GLP-1 RA on CV outcomes in adults with T2D treated with or without SGLT2i, a study [81] included a post hoc analysis of the Harmony Outcomes trial, a CVOT of albiglutide by background SGLT2i use. In addition, a trial-level meta-analysis of the Harmony Outcomes trial and the AMPLITUDE-O trial (efpeglenatide) was performed, combining the treatment effect estimates according to SGLT2i use. The results evidenced that, in patients with T2D and CVD, GLP-1 RA reduced CV events independently of SGLT2i use (P for interaction = 0.7 for MACE in the post hoc analysis; the HRs for MACE in the meta-analysis were 0.78 [95% CI 0.49 to 1.24] with SGLT2i and 0.77 [95% CI 0.76 to 0.92] without SGLT2i, P for interaction = 0.95). These findings suggest that combining GLP-1 RA with SGLT2i may further reduce CV risk.

#### R24

In adults with T2D and clinical ASCVD, who either use SGLT2i or GLP-1 RA and HbA1c remains above the target, dual therapy with AD1 plus metformin IS RECOMMENDED to improve glycemic control.
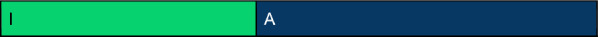


### Summary of evidence

- This panel did not find studies that evaluate sequential therapy using metformin as an add-on baseline therapy with any AD1. Notwithstanding, there is evidence about using AD1 as an add-on baseline therapy with metformin. In a network meta-analysis [82], the change in HbA1c level in patients receiving metformin-based background therapy varied from – 0.63% to – 0.51% with SGLT2i and from – 1.33% to – 0.43% with GLP– 1 RA.

#### R25

In adults with T2D and clinical ASCVD, who use SGLT2i or GLP-1 RA, and HbA1c is still above the target, dual therapy with 2 AD1 SHOULD BE CONSIDERED to improve glycemic control.
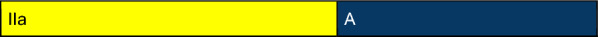


### Summary of evidence

- A systematic review and meta-analysis [83] of 7 RCTs (data from 1,913 patients, baseline HbA1c level 8-9.3%) compared the combination of GLP-1 RA plus SGLT2i vs. either agent alone to existing therapy. The combination therapy improved HbA1c (primary outcome) vs. GLP-1 RA (– 0.61%, 95% CI – 1.09 to – 0.14) and SGLT2i (– 0.85, 95% CI – 1.19 to – 0.52).

#### R26

In adults with T2D, clinical ASCVD and HbA1c above the target despite dual therapy, triple therapy with metformin and a combination of two AD1 (SGLT2i and GLP-1 RA) IS RECOMMENDED to improve glycemic control and further reduce cardiovascular events.
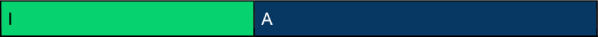


### Summary of evidence

- See the summaries of evidence for recommendations 23 and 25.

#### R27

In adults with T2D, ASCVD, and HbA1c above the target despite dual therapy, triple therapy including one AD (pioglitazone, second-generation sulfonylureas or DPP-4i) or IBT with at least one AD1 MAY BE CONSIDERED to improve glycemic control.
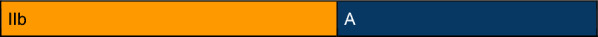


### Summary of evidence

- The efficacy and safety of DPP-4i and pioglitazone in improving hyperglycemia in patients with ASCVD are well established in the TECOS [84] (sitagliptin), SAVOR-TIMI 53 [85] (saxagliptin), CARMELINA [86] (linagliptin), and PROactive [87] (pioglitazone) trials. In addition, the efficacy and safety of sulfonylureas in patients with ASCVD were confirmed in CAROLINA [55] (glimepiride) and TOSCA.IT [56] (glimepiride) and ADVANCE [57] (gliclazide MR), as well as in a meta-analysis of RCTs.
- A meta-analysis [88] and risk–benefit assessment of pioglitazone were conducted, including studies that compared pioglitazone with a control (antidiabetic agents without pioglitazone) in patients with either established CVD or high CV risk. The use of pioglitazone compared to a control group that did not use it resulted in a 14% and 23% significant reduction in odds of major adverse cardiac events (MACE: Mantel–Haenszel odds ratio [MH-OR] 0.86, 95% CI 0.75 to 0.98), and stroke (MH-OR 0.77, 95% CI 0.60 to 0.99), respectively. The number needed to treat (NNT) for the reduction in MACE and stroke was 80 and 151, respectively. Notwithstanding, pioglitazone significantly increased the odds of HF (MH-OR 1.47, 95% CI 1.26 to 1.71) and hHF (MH-OR 1.48, 95% CI 1.21 to 1.81). The number needed to harm (NNH) for HF and hHF were 34 and 44, respectively, making these findings clinically significant. The authors concluded that pioglitazone should only be reserved for treating high CV risk or established CVD.
- The CV safety profile and HF risk of vildagliptin were evaluated in a retrospective meta-analysis [89] of prospectively adjudicated CV events, including trials in high-risk patients with T2D. Patient-level data from 17,446 patients were pooled from 40 double-blind, randomized, controlled phase III and IV vildagliptin studies. The primary endpoint was the occurrence of MACE (MI, stroke, and CV death). Vildagliptin was not associated with an increased risk of adjudicated MACEs vs. comparators (Mantel–Haenszel risk ratio [MH-RR] 0.82, 95% CI 0.61 to 1.11). Moreover, there was no significant increased risk of HF events in vildagliptin-treated patients (MH-RR 1.08, 95% CI 0.68 to 1.70).

## Management of antidiabetic therapy in adults with T2D and heart failure (HF)

**Fig. 6:**
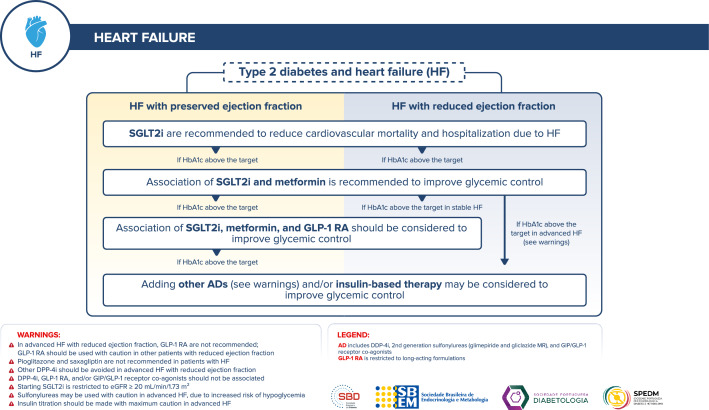
Management of antidiabetic therapy in adults with T2D and HF

Figure 6 depicts the approach to managing antidiabetic therapy in adults with T2D and HF.

### R28

In adults with T2D and HF, therapy with SGLT2i IS RECOMMENDED to reduce CV mortality and hHF and to improve glycemic control.
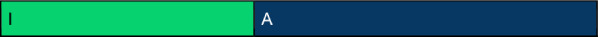


### Summary of evidence

- In a systematic review and meta-analysis [47] of 6 CVOTs of SGLT2i, including data from 46,969 patients with T2D, SGLT2i reduced the risk of CV death or hHF by 22% (HR 0.78, 95% CI 0.73 to 0.84), with a similar benefit in patients with and without HF history. In addition, SGLT2i reliably reduces the hospital admission rate for HF regardless of existing ASCVD or HF history.
- In a meta-analysis [90] of 5 RCTs including 21,947 participants with HF (with or without T2D), SGLT2i reduced the risk of composite CV death or hHF (HR 0.77, 95% CI 0.72 to 0.82), CV death (0.87, 95% CI 0.79 to 0.95), and ACM (0.92, 95% CI 0.86 to 0.99). These outcomes were consistent in trials of HF with reduced ejection fraction (HFrEF) and HF with preserved ejection fraction (HFpEF) and across all five trials.

#### R29

In adults with T2D and HF, whose HbA1c remains above target despite therapy with SGLT2i, dual therapy by adding metformin IS RECOMMENDED to improve glycemia control.
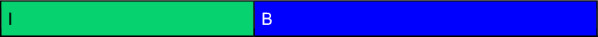


### Summary of evidence

- There are no RCTs evaluating the effects of metformin on glycemic control, specifically in patients with T2D and HF. Notwithstanding, observational evidence suggests that metformin is safe and associated with decreased mortality in patients with this profile.
- A 9-year prospective observational study [91] assessed the effect of starting metformin on the prognosis of patients with newly diagnosed HF and new-onset T2D. A total of 1,519 patients were enrolled; the mean age was 71 years, 53.8% were women, and 51.3% had preserved systolic function. Over a median follow-up of 57 months, 1,045 patients (68.8%) died, and 1,344 (88.5%) were hospitalized for decompensation of HF. There were no cases of lactic acidosis attributable to metformin use. Metformin was associated with decreased mortality (HR 0.85, 95% CI 0.82 to 0.88), driven by lower CV mortality (HR 0.78, 95% CI 0.74 to 0.82), as well as a lower hospitalization rate (HR 0.81, 95% CI 0.79 to 0.84).
- Metformin treatment in advanced HFrEF patients with T2D is associated with better outcomes by mechanisms beyond improving glycemic control. In a prospective observational study [92], propensity score-matched, including 847 stable patients with advanced HFrEF (67.7% New York Heart Association [NYHA] III/IV, left ventricular ejection fraction [LVEF] 23.6 ± 5.8%) followed for a median of 3.1 years, the subgroup of patients treated with metformin (22.9% of patients with T2D in the study) had better event-free survival even after adjustment for brain natriuretic peptide (BNP), BMI, and eGFR (HR 0.70, 95% CI 0.50 to 0.98, P = 0.035). No significant interaction was found between metformin therapy and NYHA functional class, LVEF, right ventricular dysfunction grade, BNP level, eGFR, renin–angiotensin–aldosterone system blockade, beta-blocker treatment, presence of implantable cardioverter/defibrillator, or cardiac resynchronization therapy (P for interaction ≥ 0.20).
- In an observational study [93] of 5,852 patients with HF, metformin prescription was independently associated with reduced risk of composite mortality/hHF at 12 months (HR 0.81, 95% CI 0.67 to 0.98, P = 0.03).

#### R30

In adults with T2D and heart failure with preserved ejection fraction (HFpEF) whose HbA1c remains above target despite dual therapy with metformin and SGLT2i, triple therapy by adding GLP-1 RA is safe and SHOULD BE CONSIDERED to improve glycemic control.
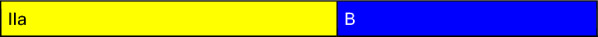


### Summary of evidence

- This panel did not find studies addressing the effect of GLP-1 RA on HF outcomes in T2D patients with HFpEF. Therefore, the following data refers to the impact of GLP-1 RA on HF-related outcomes in patients with T2D, with or without CVD.
- GLP-1 RA reduced the risk of hHF or CV death among patients without HF. In a meta-analysis [94] of 7 RCTs (data from 54,092 adults with T2D; 84% without HF, of whom 8,460 using GLP-1 RA), GLP-1 RA reduced the risk of hHF or CV death (HR 0.84, 95% CI 0.76 to 0.92) and ACM (HR 0.85, 95% CI 0.79 to 0.92).
- In a meta-analysis [95] of 7 CVOTs, including data from 56,004 adults with T2D, with or without established CVD, GLP-1 RA treatment reduced hospital admission for HF by 9% (0.91, 0.83 to 0.99; P = 0.028).
- To assess the impact of GLP-1 RA on HF or hHF in patients with T2D, a systematic review [96] included 21 RCTs (n = 18,270) and 4 observational studies (n = 111,029). In 20 RCTs, there was a lower incidence of HF with GLP-1 RA vs. control (OR 0.62, 95% CI 0.31 to 1.22). Three cohort studies evaluating GLP-1 RA vs. different comparators provided evidence that GLP-1 RA does not increase the incidence of HF. One RCT provided evidence that GLP-1 RA was not associated with hHF. The conclusion was that GLP-1 RA does not increase the risk of HF or hHF among people with T2D.

#### R31

In adults with T2D and HFpEF whose HbA1c remains above target despite dual therapy with metformin and SGLT2i, triple therapy by adding DPP-4i other than saxagliptin MAY BE CONSIDERED to improve glycemic control.
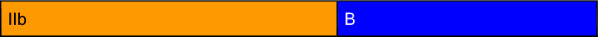


### Summary of evidence

- In a meta-analysis [97] of 4 CVOTs to assess the effects of DPP-4i on CV events (including studies with sitagliptin, alogliptin, saxagliptin, and linagliptin), the pooled analysis resulted in a neutral effect on MI, stroke, and the combination of MI plus stroke, CV death, and hHF. DPP-4i were neutral as far as all aspects of CV outcomes. Notably, in SAVOR-TIMI 53, saxagliptin increased the risk of hHF (see recommendation 36).
- The CV safety profile and HF risk of vildagliptin were evaluated in a retrospective meta-analysis [89] of prospectively adjudicated CV events, including trials in high-risk patients with T2D, such as those with congestive HF and moderate to severe renal impairment. Patient-level data from 17,446 patients were pooled from 40 double-blind, randomized, controlled phase III and IV vildagliptin studies. Assessments of the individual HF events (requiring hospitalization or new onset) were secondary endpoints. Confirmed HF events were reported in 41 (0.43%) vildagliptin-treated patients and 32 (0.45%) comparator-treated patients (RR 1.08, 95% CI 0.68 to 1.70).

#### R32

In adults with T2D, HFpEF, and HbA1c above target despite triple therapy (metformin, SGLT2i, and GLP-1 RA), adding IBT MAY BE CONSIDERED to improve glycemic control.
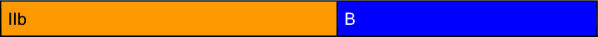


### Summary of evidence

- Although this panel did not find RCTs addressing the safety of insulin in patients with clinically established HF or at high risk of HF, there is an agreement that adding IBT may be considered a safe option to improve glycemic control whenever HbA1c target is not reached despite triple therapy, in patients with stable HF. This panel highlights, however, that close monitoring is advisable in patients with advanced HF.
- A sub-analysis of the ORIGIN trial [98] showed that glargine U100 has a neutral effect on both initial and recurrent hHF. The trial randomized 12,537 patients with prediabetes or diabetes at high CV risk to either glargine U100 or placebo. People with more severe HF (NYHA III/IV) were excluded. There were no differences between groups in hHF (HR 0.90, 95% CI 0.77 to 1.05) over the 2.5 years of follow-up.
- The ORIGINALE study [99] measured the post-trial effects of insulin glargine U100 for an additional 2.7 years. Of 12,537 randomized participants, post-trial data were analyzed for 4718 allocated initially to insulin glargine U100 (2351) vs. standard care (2,367). From randomization to the end of post-trial follow-up, no differences were found between groups in hHF (1958 vs. 1,910 events; HR 1.03, CI 95% 0.97 to 1.10, P = 0.38).
- The DEVOTE trial [100] was a treat-to-target, double-blind CVOT in 7,637 adults with T2D and high CV risk, randomized to insulin degludec or glargine U100. The primary endpoint of this secondary analysis was time to the first hHF. Severe hypoglycemia was adjudicated. Overall, 372 (4.9%) patients experienced hHF (550 events). There was no significant difference in the risk of hHF between treatments (HR 0.88, 95% CI 0.72 to 1.08, P = 0.227). Prior HF was the strongest predictor of future hHF events (HR 4.89, 95% CI 3.9 to 6.4, P < 0.0001). In patients with T2D and high CV risk, there were no treatment differences in terms of hHF.

#### R33

In adults with T2D and stable HFrEF, in whom HbA1c is above target despite dual therapy, the association of GLP-1 RA MAY BE CONSIDERED to improve glycemic control.
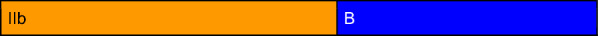


### Summary of evidence

- A meta-analysis [94] of 7 RCTs included 54,092 patients with T2D (16% with HF history; n = 8,460). Among the subgroup of patients without HF, GLP-1 RA reduced the risk of hFH or CV death (HR 0.84, 95% CI 0.76 to 0.92) and ACM (HR 0.85, 95% CI 0.79 to 0.92). In addition, a reduction of ASCVD events was observed regardless of HF history. However, GLP-1 RA did not reduce the composite of hHF or CV death (HR 0.96, 95% CI 0.84 to 1.08) or ACM (HR 0.98, 95% CI 0.86 to 1.11) in the subgroup of patients with HF history.

#### R34

In advanced heart failure with reduced ejection fraction (HFrEF), GLP-1 RA is not recommended, due to possible increases in the risk of cardiac adverse events, including hHF and all-cause mortality.
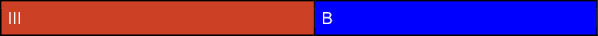


### Summary of evidence

- In the FIGHT trial [101], which included 300 patients with advanced HFrEF (hospitalization in the last 14 days; 59% with T2D; median LVEF of 25%) followed for 180 days, treatment with liraglutide did not reduce the primary endpoint of a global rank score of time to death, time to re-hospitalization for HF, and time-averaged proportional change in NT-proBNP. In a post hoc analysis of the totality of events (first and recurring), there was a trend towards increased risk with liraglutide of total HF hospitalizations or ACM (96 vs. 143 events, incidence rate ratio [IRR] 1.41, 95% CI 0.98 to 2.04, P = 0.064) and total arrhythmias (21 vs. 39, IRR 1.76, 95% CI 0.92 to 3.37, P = 0.088). Actual prespecified events of interest were increased with liraglutide vs. placebo (196 vs. 295, IRR 1.43, 95% CI 1.06 to 1.92, P = 0.018). Total hHF or ACM risk with liraglutide was higher among NYHA III/IV (IRR 1.86, 95% CI 1.21 to 2.85) and patients with T2D.
- In the LIVE trial [102], which included 241 patients with stable HFrEF, liraglutide did not improve left ventricular systolic function. It was associated with increased heart rate and more cardiac severe adverse events (10% in patients treated with liraglutide vs. 3% in the placebo group, P = 0.04).
- In a post hoc analysis of the EXSCEL trial [103], exenatide significantly increased the risk of hHF in patients with an LVEF < 40% but not in those with LVEF ≥ 40%.
- A meta-analysis [104] of the FIGHT trial and the subgroup with LVEF < 40% in the EXSCEL trial showed that GLP-1 RA increased the risk of hHF in those with reduced ejection fraction (OR 1.49, 95% CI 1.05 to 2.10).

#### R35

In adults with T2D and HF, initiating sulfonylureas MAY BE CONSIDERED with care due to a possible increase in mortality risk and new hospitalization in patients with recent hospitalizations due to HF.
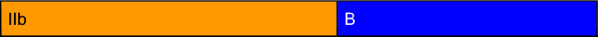


### Summary of evidence

- In an observational study [93] of 5,852 Medicare beneficiaries patients hospitalized for HF and not prescribed metformin or sulfonylurea before admission, sulfonylurea initiation within 90 days of discharge was associated with increased risk of mortality (HR 1.24, 95% CI 1.00 to 1.52, P = 0.045) and hHF (HR 1.22, 95% CI 1.00 to 1.48, P = 0.050) at 12 months, regardless of ejection fraction, as compared with patients not prescribed therapy.
- An observational study [105] investigated if ACM was associated with sulfonylureas in patients with HF. Patients hospitalized for the first time due to HF, alive 30 days after discharge, on monotherapy with a specific type of sulfonylureas were followed for a mean of 744 days. There were 1097 patients on glimepiride; 1031 on glibenclamide (glyburide); 557 on glipizide; 251 on gliclazide; and 541 on tolbutamide. During the observation period, 2242 patients (64%) died. Compared to gliclazide, which was defined as the reference, the risk of death was similar among all types of sulfonylureas: glimepiride (HR 1.10, 95% CI 0.92 to 1.33), glibenclamide (HR 1.12, 95% CI 0.93 to 1.34), glipizide (HR 1.14, 95% CI 0.93 to 1.38), and tolbutamide (HR 1.04, 95% CI 0.85 to 1.26). Significant differences in mortality risk among sulfonylureas in patients with HF were unlikely.

#### R36

Saxagliptin and pioglitazone ARE NOT RECOMMENDED in patients with HF due to the increased risk of worsening HF.
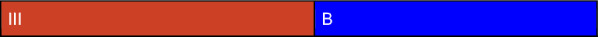


### Summary of evidence

- In the SAVOR-TIMI 53 trial [85], T2D adults at risk of CV events (n = 16,492) were randomly assigned to receive saxagliptin or placebo and followed for a median of 2.1 years. The primary efficacy and safety endpoint was the classic MACE. There were more hHF in the saxagliptin group vs. the placebo group (3.5% vs. 2.8%; HR 1.27, 95% CI 1.07 to 1.51, P = 0.007). The NNH was 143, with HF occurring early in the first year of treatment. Patients with high NT-proBNP levels, CKD, or previous HF were at increased risk.
- A meta-analysis [88] and risk–benefit assessment of pioglitazone was conducted, including studies that compared pioglitazone with a control (antidiabetic agents without pioglitazone) in patients with either established CVD or having high CV risk. The use of pioglitazone compared to the control group resulted in a 14% and 23% significant reduction in odds of MACE (MH-OR 0.86, 95% CI 0.75 to 0.98) and stroke (MH-OR 0.77, 95% CI 0.60 to 0.99), respectively. The NNT for the reduction in MACE and stroke was 80 and 151, respectively. Notwithstanding, pioglitazone significantly increased the odds of HF (MH-OR 1.47, 95% CI 1.26 to 1.71) and hHF (MH-OR 1.48, 95% CI 1.21 to 1.81). The NNH for HF and hHF were 34 and 44, respectively, making these findings clinically significant. Therefore, the authors concluded that pioglitazone should be reserved for treating T2D with high CV risk or established CVD only in selected patients where other antidiabetics are precluded and not routinely.

## Management of antidiabetic therapy in adults with T2D and kidney disease (DKD)

**Fig. 7:**
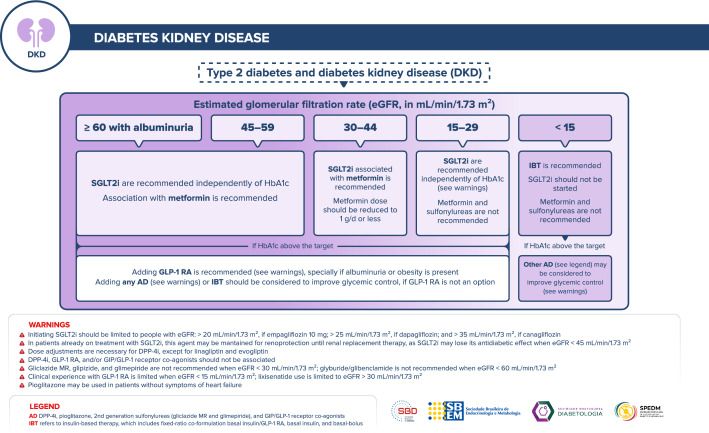
Management of antidiabetic therapy in adults with T2D and DKD

Figure 7 depicts the approach to managing antidiabetic therapy in adults with T2D and DKD.

### R37

In adults with T2D and eGFR ≥ 60 mL/min/1.73 m^2^ plus albuminuria (≥ 200 mg/g) or eGFR 30–59 mL/min/1.73 m^2^, (with or without albuminuria), dual therapy with SGLT2i plus metformin IS RECOMMENDED to attenuate long-term renal function loss, prevent end-stage renal disease, reduce mortality due to renal causes, and to improve glycemic control.
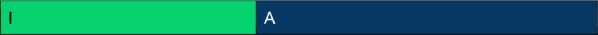


### Summary of evidence

- A systematic review and meta-analysis [106] of SGLT2i included 13 trials, with at least six months of duration, involving 90,409 adults (82.7% with T2D). The primary efficacy outcome was kidney disease progression (sustained ≥ 50% decrease in eGFR from randomization, a sustained low eGFR, end-stage kidney disease [ESKD], or death from kidney failure). Mean baseline eGFR ranged from 37–85 mL/min/1.73 m^2^. Compared with a placebo, allocation to an SGLT2i reduced the risk of kidney disease progression by 37% (RR 0.63, 95% CI 0.58 to 0.69), with similar RRs between patients with and without diabetes.
- A meta-analysis [107] of 27 studies (data from 7,363 adults with T2D and mild to moderate CKD treated with SGLT2i) demonstrate that, beyond HbA1c reduction (– 0.29%, 95% CI – 0.39 to – 0.19), SGLT2i improve blood pressure, body weight, and albuminuria. Furthermore, SGLT2i attenuated the annual decline in eGFR slope (placebo-subtracted difference of 1.35 mL/min/1.73 m^2^/year, 95% CI 0.78 to 1.93) and reduced the risk of the composite renal outcome (HR 0.71, 95% CI 0.53 to 0.95). No other additional safety concerns when SGLT2i in individuals with CKD were observed.
- This panel considered that SGLT2i might be used along with metformin in patients with CKD (eGFR ≥ 30 mL/min/1.73 m^2^) to improve glycemic control. In the CREDENCE trial [108] (canagliflozin), 57.8% of the participants were on background therapy with metformin without interfering with renal benefits.
- A meta-analysis [76] of 6 RCTs of SGLT2i, enrolling 51,743 participants, reported kidney or mortality outcomes by baseline metformin use. Background metformin therapy varied from 21% in DAPA-HF to 82% in DECLARE-TIMI 58. The HRs for the composite effect of worsening kidney function, ESKD, or kidney death were 0.58 (95% CI 0.48 to 0.69) with metformin and 0.63 (95% CI 0.48 to 0.83) without metformin (P for interaction = 0.62).

#### R38

In adults with T2D and albuminuria 30–200 mg/g, SGLT2i IS RECOMMENDED to attenuate renal function loss, prevent ESRD, and reduce mortality due to renal causes.
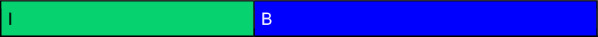


### Summary of evidence

- Subgroup analysis in a meta-analysis [109] of CV or kidney outcome trials of SGLT2i (data from 38,723 participants) reported effects on primary kidney outcomes (defined as substantial loss of kidney function, ESKD, or death due to kidney disease) in people with T2D according to the levels of albuminuria. The outcomes were stratified in subgroups according to baseline albuminuria categories: < 30 mg/g (RR 0.46, 95% CI 0.33 to 0.63, P = 0.0001); 30-300 mg/g (RR 0.69, 95% CI 0.47 to 1.00, P = 0.051), and > 300 mg/g (RR 0.52, 95% CI 0.38 to 0.69, P < 0.0001). Renoprotection was consistent across studies irrespective of baseline albuminuria (P for trend = 0.66).

#### R39

In adults with T2D and albuminuria, GLP-1 RA SHOULD BE CONSIDERED to attenuate the albuminuria progression and improve glycemic control.
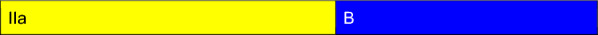


### Summary of evidence

- A systematic review and meta-analysis [110] compared the effect of GLP-1 RA and SGLT2i in kidney outcomes, including data from 8 trials (77,242 patients; 55.6% with GLP-1 RA and 44.4% with SGLT2i). GLP-1 RA reduced the risk of progression of kidney disease (HR 0.82, 95% CI 0.75 to 0.89, P < 0.001), which was exclusively dependent on albuminuria.

#### R40

Whenever HbA1c is above target despite dual therapy in T2D adults with eGFR ≥ 60 mL/min/1.73 m^2^ plus albuminuria (≥ 200 mg/g) or with eGFR 30–59 mL/min/1.73 m^2^, triple therapy with metformin, SGLT2i, and GLP-1 RA IS RECOMMENDED to reduce renal outcomes and to improve glycemic control.
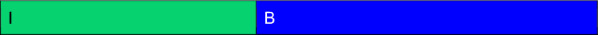


### Summary of evidence

- Sensitivity analysis of the REWIND trial [111] showed a reduced incidence of eGFR decline ≥ 40% and ≥ 50% (HR 0.70, 95% CI 0.57 to 0.85, P = 0.0004 and HR 0.56, 95% CI 0.41 to 0.76, P = 0.0002, respectively), thus supporting the hypothesis that dulaglutide may preserve kidney function. In this trial, 81% were on metformin, and 45% were on sulfonylurea.
- The AWARD-10 [61], a 24-week phase 3b RCT, placebo-controlled, assessed the safety and efficacy of the addition of dulaglutide to the ongoing treatment regimen in patients whose T2D was inadequately controlled with SGLT2i, with or without metformin. A total of 424 patients were randomized to dulaglutide 1.5 mg (n = 142), dulaglutide 0.75 mg (n = 142), and placebo (n = 140). The reduction in HbA1c at 24 weeks was more significant in patients receiving dulaglutide vs. placebo (dulaglutide 1.5 mg: – 1.34%, dulaglutide 0.75 mg: – 1.21%, placebo: – 0.54%; P < 0.0001 for both groups vs. placebo). Serious adverse events were reported for 5 (4%) participants in the dulaglutide 1.5 mg group, 3 (2%) in the dulaglutide 0.75 mg group, and 5 (4%) in the placebo group. Dulaglutide as an add-on treatment to SGLT2i, with or without metformin, resulted in significant and clinically relevant improvements in glycemic control, with acceptable tolerability consistent with dulaglutide's established safety profile.

#### R41

In adults with T2D, eGFR ≥ 60 mL/min/1.73 m^2^ plus albuminuria (≥ 200 mg/g) or eGFR 30–59 mL/min/1.73 m^2^ independently of albuminuria and HbA1c above target despite dual therapy, triple therapy with metformin, SGLT2i and an alternative AD (replacing GLP-1 RA) MAY BE CONSIDERED to improve glycemic control.
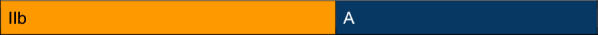


### Summary of evidence

Adding DPP-4i:

- Linagliptin: The CARMELINA trial [86], a multicenter non-inferiority RCT, evaluated linagliptin vs. placebo in 6,979 adults with T2D and high CV and renal risks during a median follow-up of 2.2 years. Participants had either an eGFR between 45 and 75 mL/min/1.73 m^2^ plus UACR > 200 mg/g or an eGFR between 15 and 45 mL/min/1.73 m^2^ regardless of UACR. Around 40% of patients had dual therapy at baseline and received triple therapy. The mean eGFR was 54.6 mL/min/1.73 m^2^, and most patients had eGFR between 30 and 60 mL/min/1.73 m^2^. The primary outcome (MACE) was similar in both groups (HR 1.02, 95% CI 0.89 to 1.17), indicating safety (P < 0.001), as was the renal outcomes (ESKD, death due to renal failure, or a sustained eGFR decline ≥ 40%; HR 1.04, 95% CI 0.89 to 1.22, P = 0.62). The rates of adverse events, serious adverse events, and adverse events leading to discontinuation were not different between linagliptin and placebo. Linagliptin is considered safe for renal failure.
- Sitagliptin: The safety of sitagliptin in adults with T2D and moderate to severe CKD (eGFR < 50 mL/min/1.73 m^2^, including adults with ESKD on dialysis) was assessed in a 54-week, randomized, double-blind, parallel-group study [112]. Participants in the sitagliptin group (n = 65) and placebo group (n = 26) had baseline HbA1c between 6.5 and 10%. At 54 weeks, patients continuously treated with sitagliptin had a mean change from baseline in HbA1c of -0.7% (95% CI – 0.9 to – 0.4).
- The COMPOSIT-R trial [113] included 614 T2D adults with CKD (eGFR 60–90 mL/min/1.73 m^2^) and HbA1c of 7–9.5%, who were on metformin alone or metformin plus sulfonylurea. Participants were randomized to sitagliptin or dapagliflozin. The mean eGFR at baseline was 79.4 ± 11.3 mL/min/1.73 m^2^. Around 30% of patients were on dual therapy. After 24 weeks, the change in HbA1c from baseline was more remarkable with sitagliptin (– 0.51%, 95% CI – 0.60 to – 0.43) than dapagliflozin (– 0.36%, 95% CI – 0.45 to – 0.27); the difference was – 0.15% (95% CI – 0.26 to – 0.04) to sitagliptin vs. dapagliflozin (P = 0.006). Overall, adverse events were similar between groups. No serious adverse events or deaths were reported with triple therapy.

Adding pioglitazone:

- A meta-analysis [114] evaluated the efficacy and safety of thiazolidinediones, including pioglitazone and rosiglitazone, in treating T2D patients with renal impairment. Nineteen RCTs were included, covering 1,818 participants, with a mean age ranging from 43.4 to 71.1 years, mean baseline HbA1c of 6.9 to 9.2%, and mean follow-up of 24 weeks. Of the 19 RCTs, one trial (5.3%) enrolled patients who have undergone renal transplantation, five (26.3%) enrolled dialysis patients, and 13 (68.4%) included patients with mild to moderate renal impairment. Fourteen trials (73.7%) used pioglitazone as the intervention, four (21.1%) used rosiglitazone, and one (5.3%) used both. Thiazolidinediones were not associated with an increased risk of ACM (RR 0.40, 95% CI 0.08 to 2.01) and did not increase the risk of HF (RR 0.64, 95% CI 0.15 to 2.66, I^2^ 0%). Compared to the control, however, they significantly increased the risk of edema (RR 2.96, 95% CI 1.22 to 7.20).
- A small efficacy and tolerability trial [115] randomized 93 adults with T2D and CKD (eGFR < 60 mL/min/1.73 m^2^ or albuminuria, of whom 30% were stage II, 32% were stage III, and 27% were stage IV) to pioglitazone 15 mg (standard-dose) or 7.5 mg (low-dose) for 24 weeks. The mean change in HbA1c did not differ between the standard-dose and low-dose groups (-1.1 ± 1.6 and -1.4 ± 1.5, P = 0.543, respectively). Standard-dose pioglitazone was associated with greater increases in body weight, fat mass, total body mass, total body water, and extracellular water compared to the low-dose regimen. Compared to patients in the low-dose group, those in the standard-dose group experienced significant, though modest, weight gain (3.5 ± 3.2 vs. 0.2 ± 4.4 kg; mean difference between groups 3.3 kg, 95% CI 1.3 to 5.2). No significant adverse effects (including hypoglycemia, congestive HF, and abnormal liver function) were identified. This study indicated that low-dose pioglitazone has similar efficacy while promoting less weight gain than standard-dose pioglitazone in patients with CKD.

Adding sulfonylureas:

- The safety of sulfonylureas was evaluated in the CAROLINA trial [55], a head-to-head, active-controlled, randomized trial that assessed the impact of linagliptin vs. glimepiride on CV outcomes in high-risk patients (many with CKD). The eGFR (mL/min/1.73 m^2^) was 30–59 in 19% and 15–29 in 0.4% of participants. The primary outcome was time to the first occurrence of a MACE event to establish the noninferiority of linagliptin vs. glimepiride. A primary outcome event occurred in 356 of 3,023 patients (11.8%) in the linagliptin group and 362 of 3,010 (12%) in the glimepiride group (HR 0.98, 95% CI 0.84 to 1.14; P < 0.001 for non-inferiority). Thus, linagliptin met the noninferiority criterion but not the superiority criterion (P = 0.76). The incidence of adverse events was similar in the linagliptin and glimepiride groups. Hypoglycemia, as expected, was increased in the glimepiride group: 10.6% in the linagliptin group and 37.7% in the glimepiride group (HR 0.23, 95% CI 0.21 to 0.26).

#### R42

In adults with T2D, eGFR ≥ 60 mL/min/1.73 m^2^ plus albuminuria (≥ 200 mg/g) or eGFR 30–59 mL/min/1.73 m^2^ independently of albuminuria and HbA1c above target despite triple therapy, quadruple therapy including metformin, SGLT2i, GLP-1 RA and a fourth AD or IBT MAY BE CONSIDERED to improve glycemic control.
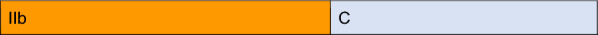


### Summary of evidence

- Although this panel did not find significant efficacy evidence for QUADRUPLE therapy in T2D patients with mild to moderate renal failure, it may be considered that this strategy is necessary to lower blood glucose in some patients. Furthermore, it is reasonably safe in stage 3 CKD (eGFR 30–60 mL/min/1.73 m^2^), when most agents can be used, provided that their dosages are adjusted when appropriate. Special attention is warranted with metformin, which should be replaced when the eGFR falls below 30 mL/min/1.73 m^2^. Sulfonylureas also demand caution due to this population's increased risk of hypoglycemia.

#### R43

In adults with T2D, eGFR < 30 mL/min/1.73 m^2^, and HbA1c mildly above target, either DPP-4i or GLP-1 RA (if eGFR 15–30 mL/min/1.73 m^2^) MAY BE CONSIDERED to improve glycemic control.
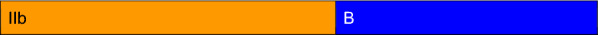


### Summary of evidence

Adding DDP-4i:

- The DPP-4i class (sitagliptin, vildagliptin, alogliptin, saxagliptin, linagliptin, and evogliptin) was also tested in small studies in T2D patients undergoing hemodialysis, and safety should be confirmed in larger studies.
- In a small trial [116], 64 patients with T2D were randomized to sitagliptin (in the reduced dosage of 25 mg/d) and 65 to glipizide 2.5 mg/d. There were 28 patients (43%) with eGFR < 30 mL/min/1.73 m^2^. After 54 weeks, the mean reduction in HbA1c level from baseline was 0.72% (95% CI 0.95% to 0.48%) in the sitagliptin group and 0.87% (95% CI 1.11% to 0.63%) in the glipizide group. The incidence of symptomatic hypoglycemia was 6.3% in the sitagliptin group vs. 10.8% in the glipizide group (difference 4.8%, 95% CI 15.7% to 5.6%). Severe hypoglycemia did not occur in the sitagliptin group vs. 7.7% in the glipizide group (difference 7.8%, 95% CI 17.1% to 1.9%). Sitagliptin monotherapy was effective and well tolerated in patients undergoing hemodialysis.
- In a multicenter RCT [117], adults with T2D, either drug-naive or not, who had inadequate glycemic control (HbA1c 6.5–10%) and an eGFR < 30 mL/min/1.73 m^2^, were randomized to vildagliptin 50 mg/d (n = 83) or sitagliptin 25 mg/d (n = 65). After 24 weeks, the adjusted mean change in HbA1c was – 0.54% from a baseline of 7.52% with vildagliptin and – 0.56% from a baseline of 7.80% with sitagliptin (P = 0.874). Both agents were well tolerated, with overall similar safety profiles.
- In a small non-randomized safety trial [118], 16 patients with T2D undergoing hemodialysis received alogliptin 6.25 mg/d for two years. Baseline serum creatinine was 10.6 ± 1.0 mg/dL. Mean HbA1c dropped from 7.1 to 5.8% during the treatment. None of the patients exhibited significant adverse effects, such as hypoglycemia. However, one patient experienced a drug-related rash, and four withdrew from this study during treatment.
- The effects of monotherapy with linagliptin five mg/d in 21 adults with T2D undergoing hemodialysis was examined in a 6-month non-randomized trial [119]. Linagliptin was administered daily. Glycated albumin dropped from 21.3% ± 0.6% to 18% ± 0.6% over the 6-month treatment period, and body weight did not change. None of the patients experienced hypoglycemia.
- In a sub-analysis of the SAVOR-TIMI trial [120], adults with T2D at risk for CV events, randomized to saxagliptin or placebo, were stratified according to eGFR (mL/min/1.73 m^2^): > 50 (n = 13,916), 30–50 (n = 2,240), or < 30 (n = 336). After a median follow-up of 2 years, saxagliptin was like placebo for the primary outcome (MACE) and secondary composite outcomes, irrespective of renal function (all P for interactions ≥ 0.19). The relative risk of hHF with saxagliptin was similar (P for interaction = 0.43) in participants with eGFR > 50 (HR 1.23, 95% CI 0.99 to 1.55), eGFR 30–50 (HR 1.46, 95% CI 1.07 to 2.00), and eGFR < 30 (HR 0.94, 95% CI 0.52 to 1.71). In these CKD patients, the median HbA1c at one year was lower in saxagliptin-treated vs. placebo (7.1% vs. 7.7%, P = 0.002). At least one adverse event occurred in 152 (88%) saxagliptin-treated patients with renal impairment compared with 126 (77%) patients treated with placebo (P = 0.006), with no significant difference in severe adverse events.

Adding GLP-1 RA:

- Data for the use of GLP-1 RA in T2D with severe renal failure (< 30 mL/min/1.73 m^2^) are derived from subsets of more extensive trials that included a minimal number of patients, such as 2.5% in LEADER RENAL [121] (liraglutide), 2.5% in SUSTAIN-6 [78] (injectable semaglutide), and 1% in REWIND RENAL [111] (dulaglutide). Thus, data on the safety of GLP-1 RA in this population is limited.

#### R44

In adults with T2D, eGFR < 30 mL/min/1.73 m^2^, and HbA1c above target, IBT IS RECOMMENDED to improve glycemic control.
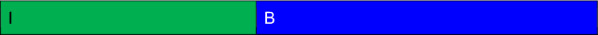


### Summary of evidence

- Glargine U100 is safe and effective in T2D patients with severe renal failure, yielding rapid HbA1c reductions with a stable half-life and longer duration of action. In a small non-randomized study [122], 89 patients with T2D and CKD (mean eGFR 34.1 ± 11.5 mL/min/1.73 m^2^), who were poorly controlled or experienced frequent hypoglycemia on oral ADs or NPH insulin, were prescribed glargine U100 at bedtime. The dose started at 0.1 units/kg and was titrated to the target. At four months of follow-up, HbA1c had declined from 8.4% ± 1.6 to 7.7% ± 1.2 (P < 0.001). BMI was unaffected (P = 0.96). Mild symptomatic hypoglycemia was experienced by 12.5% of patients, and no other adverse events were reported.
- A small single-center retrospective observational study [123] evaluating adults with T2D and CKD using basal insulin for at least 24 weeks assessed the efficacy and safety of glargine U100 (n = 35) vs. degludec (n = 37). In advanced renal failure (stage 4 CKD), there was less hypoglycemia with degludec than glargine U100 (P = 0.009), indicating that degludec may be a safer option.

#### R45

In adults with T2D and eGFR < 30 mL/min/1.73 m^2^, already on treatment with SGLT2i, it MAY BE CONSIDERED to continue the SGLT2i unless not tolerated or ESKD is initiated.
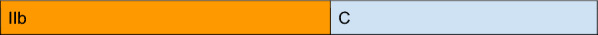


### Summary of evidence

- In the EMPA-KIDNEY trial [124], patients with CKD (eGFR 20 to < 45 mL/min/1.73 m^2^ or eGFR of 45–90 mL/min/1.73 m^2^ and UACR ≥ 200 mg/g) were randomly assigned to receive empagliflozin ten mg/d or matching placebo (n = 6,609). The primary outcome was a composite of the progression of kidney disease. During a median of 2.0 years of follow-up, progression of kidney disease occurred in 13.1% in the empagliflozin group and 16.9% in the placebo group (HR 0.72, 95% CI 0.64 to 0.82, P < 0.001). Results were consistent across the subgroups defined according to eGFR ranges, including patients with eGFR < 30 mL/min/1.73 m^2^.
- The KDIGO 2020 guideline [125] states that long-term benefits of SGLT2i regarding eGFR preservation are observed despite the initial decline and a reversible decrease after initiating SGLT2i. In the CREDENCE trial [108], canagliflozin was continued among participants whose eGFR fell below 30 mL/min/1.73 m^2^. Based on the CREDENCE protocol, it is reasonable to continue an SGLT2i even if the eGFR falls below 30 mL/min/1.73 m^2^ unless not tolerated or ESKD is initiated.

## Conclusions

The current management of antidiabetic therapy for people with T2D needs to evaluate aspects beyond glycemic control, requiring a comprehensive patient-centered approach and considering the best evidence available. All individuals with T2D must have their CV risk status stratified, the renal function assessed, and BMI as well as HbA1c determined before defining the use of antidiabetic agents. A personalized HbA1c target of less than 7% for most adults with T2D should be reassessed regularly (once every 12 weeks in unstable situations or at least once every 24 weeks in patients meeting goals). Non-pharmacological approaches, such as nutritional intervention, focusing on weight control, physical exercise, decreasing sitting time, improving sleep duration, stopping smoking, and stress management, are recommended during all phases of treatment, and the use of CGM should be considered, bearing in mind the cost–benefit ratio.

Metformin is the agent of choice in treatment-naïve adults recently diagnosed with T2D, without CVD or CKD, either in monotherapy or initial combination with AD1 or ADs, depending on the CV risk assessment, BMI, and HbA1c level. Notably, in adults with T2D at high or very high CV risk, AD1 is recommended for the reduction of CV events; if obesity is present, GLP-1 RA or GIP/GLP-1 receptor co-agonists (e.g., tirzepatide) should be considered, independently of HbA1c, for improving weight loss. In people whose HbA1c remains above target, dual, triple, and quadruple therapy, or IBT, should be considered to improve glycemic control. In asymptomatic adults with T2D requiring IBT, FRC insulin/GLP-1 RA should be considered (if available) over basal or basal-bolus insulin when available. Moreover, if HbA1c > 9% and severe signs or symptoms of hyperglycemia (polyuria, polydipsia, weight loss) are present, IBT must be the choice.

In adults with T2D with clinical ASCVD, AD1 is recommended to reduce CV events and CV mortality. Notwithstanding, if HbA1c remains above target, combining GLP-1 RA plus SGLT2i may be considered, followed by metformin, other ADs, or IBT to improve glycemic control. In adults with T2D and HF, therapy with SGLT2i is recommended to reduce CV mortality and hHF and to improve glycemic control, and if HbA1c remains above target despite treatment with SGLT2i, metformin is recommended, and other ADs or IBT may be considered, avoiding saxagliptin and pioglitazone. Furthermore, in advanced HFrEF, GLP-1 RA is not recommended due to the increased risk of serious cardiac adverse events, and initiating sulfonylureas is not recommended in adults with T2D and recent hHF due to the possible increased risk of mortality and new hospitalization.

In adults with T2D, DKD, and eGFR ≥ 30 mL/min/1.73 m^2^, therapy with SGLT2i is recommended, significantly to improve renal outcomes; these cut-offs of eGFR may vary according to specific SGLT2i agent (20 mL/min/1.73 m^2^, if empagliflozin 10 mg [124]; 25 mL/min/1.73 m^2^, if dapagliflozin [126]; and 35 mL/min/1.73 m^2^, if canagliflozin [108]). If HbA1c is above target, metformin is usually the second agent of choice, although GLP-1 RA should be considered if albuminuria is present to attenuate its progression and to improve glycemic control. Whenever HbA1c is above target despite dual therapy, triple therapy with metformin, SGLT2i, and GLP-1 RA is recommended to reduce renal outcomes and improve glycemic control. Suppose eGFR < 30 mL/min/1.73 m^2^, IBT is recommended, although either DPP-4i or GLP-1 RA (if eGFR 15–30 mL/min/1.73 m^2^) may be considered if HbA1c is mildly above target. In adults with T2D and eGFR < 30 mL/min/1.73 m^2^, already on treatment with SGLT2i, it may be continued unless not tolerated or ESKD is initiated. These recommendations synthesize the best evidence for managing antidiabetic therapy in people with T2D.

## Data Availability

Not applicable.

## Abbreviations

ACM: All-cause mortality
AD: Antidiabetic drug
AD1: First-line antidiabetic drugs
ASCVD: Atherosclerotic cardiovascular disease
BGM: Blood glucose meter
BMI: Body mass index
BNP: Brain natriuretic peptide
CGM: Continuous glucose monitoring
CI: Confidence interval
CIT: Conventional insulin injection therapy
CKD: Chronic kidney disease
CSII: Continuous subcutaneous insulin infusion
CV: Cardiovascular
CVD: Cardiovascular disease
CVOT: CV outcome trial
DKD: Diabetes, kidney disease
DPP-4i: Dipeptidyl peptidase-4 inhibitors
DSME: Diabetes self-management education
eGFR: Estimated glomerular filtration rate
ESKD: End-stage kidney disease
ETD: Estimated treatment difference
FPG: Fasting plasma glucose
FRC: Fixed-ratio co-formulation
GIP: Glucose-dependent insulinotropic polypeptide
GLP-1 RA: Glucagon-like peptide-1 receptor agonists
GLP-1: Glucagon-like peptide-1
HbA1c: Glycated hemoglobin
HF: Heart failure
HFpEF: Heart failure with preserved ejection fraction
HFrEF: Heart failure with reduced ejection fraction
hHF: Hospitalization for heart failure
HOMA-β: Homeostasis model assessment of β-cell function
HR: Hazard ratios
IBT: Insulin-based therapy
IRR: Incidence rate ratio
isCGM: Intermittently scanned continuous glucose monitoring
LSM: Least squares mean
LVEF: Left ventricular ejection fraction
MACE: Major adverse cardiovascular events
MDI: Multiple daily injections
MH-OR: Mantel–Haenszel odds ratio
MH-RR: Mantel–Haenszel risk ratio
MI: Myocardial infarction
MIT: Multiple insulin injections
NHANES: National Health and Nutrition Examination Survey
NNH: Number needed to harm
NNT: Number needed to treat
NYHA: New York Heart Association
OR: Odds ratio
RCT: Randomized clinical trial
RR: Relative risk
SBD: Sociedade Brasileira de Diabetes
SBEM: Sociedade Brasileira de Endocrinologia e Metabologia
SC: Subcutaneous
SGLT2i: Sodium-glucose cotransporter-2 inhibitors
SPD: Sociedade Portuguesa de Diabetologia
SPEDM: Sociedade Portuguesa de Endocrinologia, Diabetes e Metabolismo
T2D: Type 2 diabetes mellitus
UACR: Urine albumin-creatinine ratio
UK: United Kingdom
WMD: Weighted mean difference
